# Neglected Posterior Cervical Myelomeningocele in Adults: Systematic Review of the Literature and Illustrative Case

**DOI:** 10.3390/brainsci16101107

**Published:** 2026-10-14

**Authors:** Adi Ahmetspahic, Razim Mahmutagic, Bekir Rovcanin, Ibrahim Omerhodzic, Edina Dozic, Deniz Bulja, Mirza Pojskic, Kenan I. Arnautović

**Affiliations:** 1 Department of Neurosurgery, Clinical Center of University of Sarajevo, 71000 Sarajevo, Bosnia and Herzegovina; adi.ahmetspahic@ssst.edu.ba (A.A.); bekir_rovcanin@hotmail.com (B.R.); ibrahim.omerhodzic@mf.unsa.ba (I.O.); 2 Medical School, Sarajevo School of Science and Technology, 71000 Sarajevo, Bosnia and Herzegovina; edina.djozic@ssst.edu.ba (E.D.); deniz.bulja@ssst.edu.ba (D.B.); 3 Faculty of Medicine, University of Sarajevo, 71000 Sarajevo, Bosnia and Herzegovina; razim_m@outlook.com; 4 Department of Neurology, Clinical Center of University of Sarajevo, 71000 Sarajevo, Bosnia and Herzegovina; 5 Department of Radiology, Clinical Center of University of Sarajevo, 71000 Sarajevo, Bosnia and Herzegovina; 6 Department of Neurosurgery, Philipps University of Marburg, 35043 Marburg, Germany; 7 Department of Neurosurgery, University of Tennessee Health Science Center, Memphis, TN 38163, USA; kenanarnaut@yahoo.com; 8 Department of Neurosurgery, Semmes Murphey Clinic, Memphis, TN 38163, USA

**Keywords:** posterior cervical myelomeningocele, spinal dysraphism, congenital spinal anomaly, tethered cord, adult

## Abstract

**Highlights:**

**What are the main findings?**

- This systematic review identified 23 reported adults with posterior cervical cystic spinal dysraphism in 19 studies, most of them symptomatic and operated.
- We add one radiologically diagnosed C4–C5 lesion in a 62-year-old man without objective deficit, managed by observation.

**What are the implications of the main findings?**

- The evidence is sparse and prone to publication bias, and it cannot define natural history or compare surgery with observation.
- Standardised terminology and multicentre registries with long-term follow-up are needed.

**Abstract:**

**Objectives**: Adult posterior cervical spinal dysraphism, including meningocele (MC), myelomeningocele (MMC), and myelocystocele (MyCC) is exceptionally rare and remains insufficiently characterised. This study aimed to conduct the first systematic review of the literature on adult cases and report an additional illustrative case. **Methods**: A PRISMA 2020 systematic review of PubMed/MEDLINE, Web of Science and Scopus was conducted (PROSPERO CRD420261426944). Data were extracted at case level, grouped by the lesion label used by the original authors, and synthesised narratively. **Results**: Nineteen studies (18 case reports and one case series) described 23 adults (median age 24 years; 19 male): 16 MC, one MyCC and six MMC. An objective neurological abnormality was documented in 14 of 21 examined patients. Nineteen patients were operated on; among 17 with a classified outcome, ten improved, five were intact and two were stable, and none were reported worse (median follow-up 12 months). The additional case was a 62-year-old man with a radiologically diagnosed C4–C5 lesion most consistent with MMC and no objective neurological deficit, managed by observation (3 months of follow-up). **Conclusions**: The evidence is sparse, heterogeneous and prone to publication bias, and it is insufficient to establish natural history, optimal management or the relative merits of surgery and observation. Standardised terminology, multicentre registries and long-term follow-up are needed.

## 1. Introduction

Neural tube defects (NTD) arise from incomplete neural tube closure during early embryogenesis and are among the most common congenital malformations of the central nervous system, with a worldwide birth prevalence of 1–10 per 1000 live births [1]. Cervical spinal involvement remains exceptionally uncommon, accounting for only 1–5% of all NTDs [2]. Based on cyst contents, these lesions are commonly classified as meningocele (MC), myelomeningocele (MMC), and myelocystocele (MyCC). MC is characterised by a meningeal sac containing only cerebrospinal fluid (CSF), without neural tissue, or, in rudimentary MC, by a solid mass attached to the spinal cord by a fibrous stalk, whereas MMC contains neural elements, such as the spinal cord and/or nerve roots within the protruding sac. MyCC represents a dilated central canal herniating into a meningocele [3]. While periconceptional folic acid supplementation is known to reduce NTD recurrence, this recommendation postdated the birth of our patient by several decades [4]. Embryologically, the lesion has been attributed to failed separation of the neural and cutaneous ectoderm during neurulation, producing a fibroneurovascular stalk capable of tethering the spinal cord to the skin [5,6]. For limited dorsal myeloschisis (LDM), this has been termed incomplete disjunction. These are proposed mechanisms, and even the sequence of human neural tube closure remains debated [7].

Most cervical dysraphisms are repaired in infancy; adult presentations are confined to isolated reports, and the natural history of unrepaired lesions persisting into adulthood remains poorly characterised [5,8,9]. Associated abnormalities may include cord tethering, Chiari II malformation, lipomyelomeningocele, diastematomyelia, and Klippel–Feil syndrome [10]. Cervical lesions are often covered by intact epithelium [11]. Relatively preserved neurological function has been reported in some patients and has been attributed to a late and limited neurulation abnormality [12], but whether this applies to adults is unknown.

Although Ricci et al. [13] addressed anterior MC in adults and Maroufi et al. [14] reviewed saccular LDM, previously called cervical MMC, in predominantly paediatric patients, no equivalent synthesis exists for posterior cervical spinal dysraphism in the adult population. The objective of this study was to conduct, to our knowledge, the first systematic review of posterior cervical spinal dysraphism in adults. Cases reported as MC (including rudimentary MC), MMC, or MyCC were all considered, and results are reported separately by lesion group. Additionally, we present a previously undocumented case of an unrepaired C4–C5 lesion, most consistent with MMC on radiological grounds, with associated congenital anomalies.

## 2. Materials and Methods

### 2.1. Illustrative Case

An original case of an adult with a posterior cervical lesion, most consistent with MMC on radiological grounds, is included as an additional unreported case contributing to the systematic review dataset. Written informed consent for publication of clinical details and accompanying images was obtained from the patient. The diagnosis rests on computed tomography (CT) and magnetic resonance imaging (MRI) interpreted by a neuroradiologist (D.B.) and on neurological examination and electromyoneurography (EMNG) performed by a neurologist (E.Đ.). All imaging was reviewed on a dedicated diagnostic workstation (IDS7, version 25.1.15.4485; Sectra AB, Linköping, Sweden). EMNG was performed with the UltraPro S100 system (Natus Medical Incorporated, Middleton, WI, USA). The lesion was managed conservatively, so there is no operative or histopathological confirmation. The present case is reported separately from the previously published cases throughout the Results.

### 2.2. Systematic Review Methodology

#### 2.2.1. Study Design and Search Strategy

This systematic review was conducted in accordance with the Preferred Reporting Items for Systematic Reviews and Meta-Analyses (PRISMA) 2020 guidelines [15] and is registered on the International Prospective Register of Systematic Reviews (PROSPERO registration number CRD420261426944) on 17 June 2026, prior to full-text screening, quality appraisal, and data extraction; the record was amended twice (7 September 2026 and 6 October 2026) to reflect the protocol deviations. A comprehensive search was conducted across MEDLINE via PubMed (4 June 2026), the Web of Science Core Collection (4 June 2026), and Scopus (23 September 2026), without date restrictions. The PubMed and Web of Science searches were not repeated when Scopus was added. Embase was not searched because institutional access was not available, so reports indexed only in Embase may have been missed. The search strategies were developed by the authors without an information specialist or librarian. Three complementary search strategies were employed: a broad sensitivity search (Strategy A), a targeted sub-search for adult and neglected presentations (Strategy B), and a targeted sub-search for syringomyelia co-occurrence (Strategy C); full search strings for each database, including field tags, Boolean operators, controlled vocabulary, and search dates, are provided in Supplementary Table S1. Records from the three databases were exported and deduplicated with the Automated Systematic Search Deduplicator (ASySD; University of Edinburgh, Edinburgh, UK) [16] using its default settings; no manual deduplication was performed. The deduplicated records were imported into Rayyan (Rayyan Systems Inc., Cambridge, MA, USA) [17] for screening. Reference lists of all retrieved full-text articles were manually screened (backward citation searching), and citing literature for all included studies was checked (forward citation searching), to identify additional eligible studies not captured by the database search. 

#### 2.2.2. Eligibility Criteria and Study Selection

**Figure 1:**
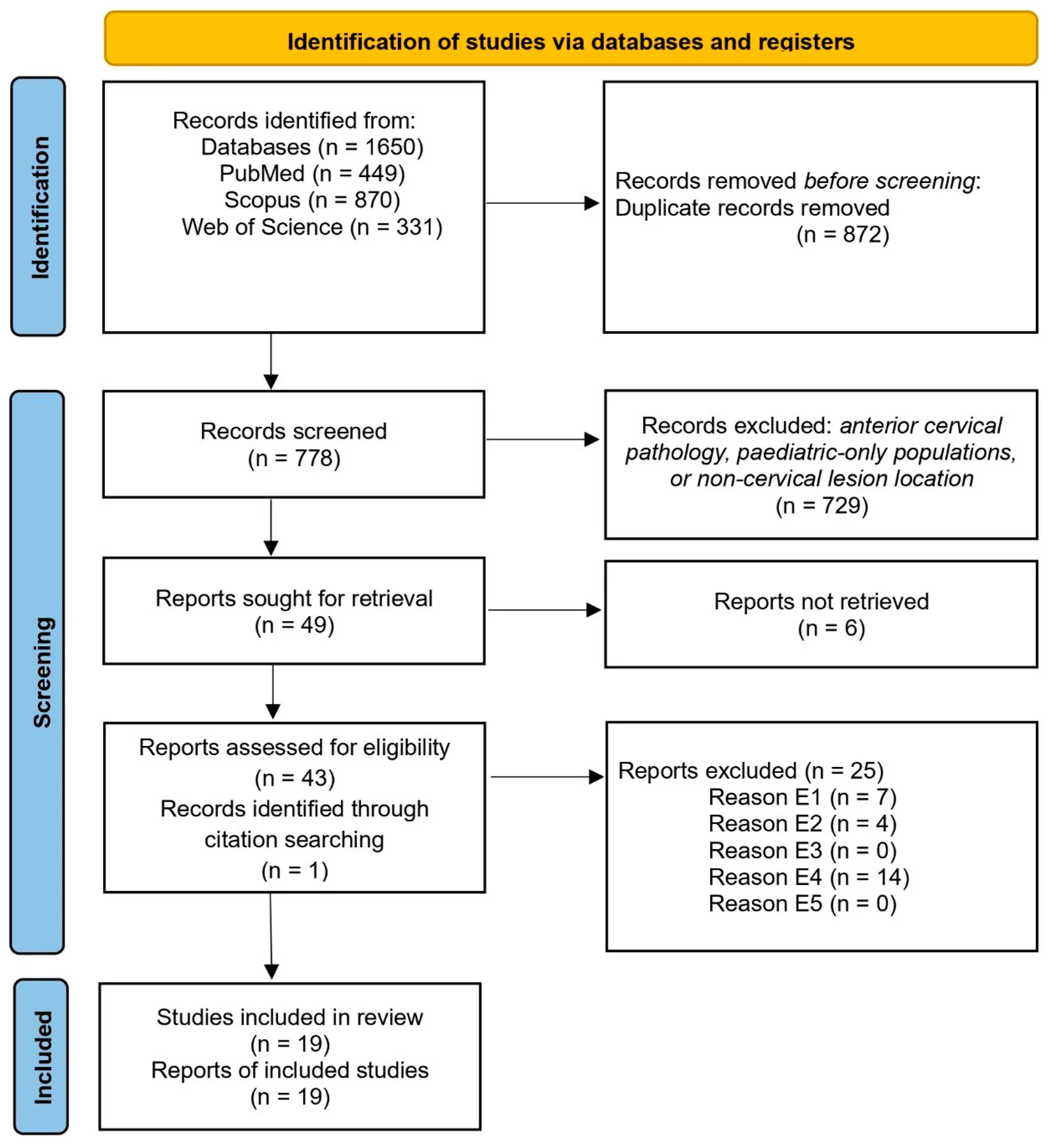
PRISMA flow diagram. Note: E1, primary diagnosis other than cervical MC, MMC or MyCC, or lesion outside the eligible cervical level (including anterior and cervicothoracic lesions); E2, ineligible study type; E4, exclusively paediatric population.

Two independent reviewers (R.M. and B.R.) screened titles and abstracts following duplicate removal; full-text articles were independently assessed by the same two reviewers against inclusion/exclusion criteria. Disagreements were resolved by a third reviewer (A.A.). Reports whose full text could not be retrieved were published before the digitisation of journal archives. Reports in languages other than English were translated into English for assessment. Lesions were grouped by the label used in the original report: MC (including rudimentary MC), MyCC (including non-terminal MyCC), and MMC (including recurrent and retethered MMC). The three groups were analysed together because they were considered related cystic dorsal lesions with overlapping terminology in the literature, without implying biological equivalence. All findings were nevertheless reported separately by group. Cervical saccular LDM was not included as a separate category because the available adult reports do not consistently provide sufficient information on the stalk, skin, and histopathological features required for definitive classification as LDM. The study selection process is shown in the PRISMA flow diagram (Figure 1, Supplementary Material PRISMA 2020 Checklist), and eligibility criteria are summarised in Table 1.

#### 2.2.3. Methodological Appraisal (JBI)

Methodological quality was assessed descriptively using the Joanna Briggs Institute (JBI) Critical Appraisal Checklist for Case Reports (8 items) [18] for single-patient studies, and the JBI Critical Appraisal Checklist for Case Series (10 items) [19] for Duz et al. [8], to characterise the methodological strengths and limitations of the included evidence (Supplementary Tables S3 and S4); appraisal was conducted independently of, and did not influence, study eligibility (Table 1). The JBI checklists are critical appraisal tools rather than validated risk-of-bias scales. Appraisal was therefore reported item by item, without a summed score, cut-off, or overall risk-of-bias category, and was not used to grade certainty of evidence. The appraisal was intended to describe reporting completeness rather than quantify the strength of evidence for prognosis or treatment effects.

#### 2.2.4. Data Extraction and Synthesis

For each included study, the following data were extracted: country of origin; patient age and sex; lesion type (MC, MMC, or MyCC) as labelled by the original authors; anatomical level; prior repair of the same lesion; presence of a syrinx; presenting symptoms and duration prior to diagnosis; associated anomalies; imaging findings; surgical management and its indication; complications (adverse events); recurrence or progression at last follow-up; and follow-up with clinical outcome. Objective neurological abnormality was defined as any abnormal finding on the neurological examination reported by the original authors (for example, weakness, sensory loss, abnormal reflexes or tone, pathological reflexes, gait or coordination disturbance, or mirror movements). Symptoms without a described examination finding were coded as not reported, and a normal examination was coded as no. Outcome categories followed the wording of the original authors: improvement of a preoperative deficit or symptom (IMP); neurologically intact before and after treatment (INT); no deterioration or recurrence without stated improvement (STB). An outcome that the authors did not report was coded as not reported (NR), and not applicable (N/A) was used only for a documented absence of intervention. Where a result is calculated from a subset of cases, the denominator is stated. Data extraction and appraisal were performed independently by two reviewers (R.M. and B.R.), and disagreements were resolved by a third reviewer (A.A.). Data were narratively synthesised and presented in tabular form (Table 2) by lesion group; the present case is reported separately from the previously published cases. Frequencies describe the reported cases and are not estimates of treatment effect or of the proportion of patients who need surgery. Meta-analysis was not performed, and publication bias was not assessed formally because the evidence consists of case reports and small series.

#### 2.2.5. Protocol Amendments

The protocol was registered on 17 June 2026 and amended twice. Amendment 1 (7 September 2026) was made in response to the editor’s comments. It added an English-language restriction, narrowed the eligibility criterion on clinical detail (I4 and E3) to require documented treatment or outcome, and added the present case as a descriptive data point. Amendment 2 (6 October 2026) was made in response to peer review. It added Scopus, widened I4 and E3 so that only demographic and clinical detail sufficient for case-level extraction was required, regardless of treatment or outcome reporting, and replaced the English-language restriction with translation of non-English reports. Exclusion criteria were also renumbered (E4, paediatric patients; E5, genetic, embryological or molecular findings only), and E1 was reworded to state explicitly that lesions extending from the cervical into the thoracic spine and primary diagnoses other than cervical MC, MMC or MyCC are not eligible, in line with criteria I1 and I3. Both amendments were made after the database searches and with knowledge of the earlier results. The amendments could therefore have changed the number and characteristics of the included studies: the narrowing in amendment 1 favoured the exclusion of conservatively managed and incidentally identified cases, and the widening in amendment 2 reverses this.

## 3. Results

### 3.1. Case Presentation

**Figure 2:**
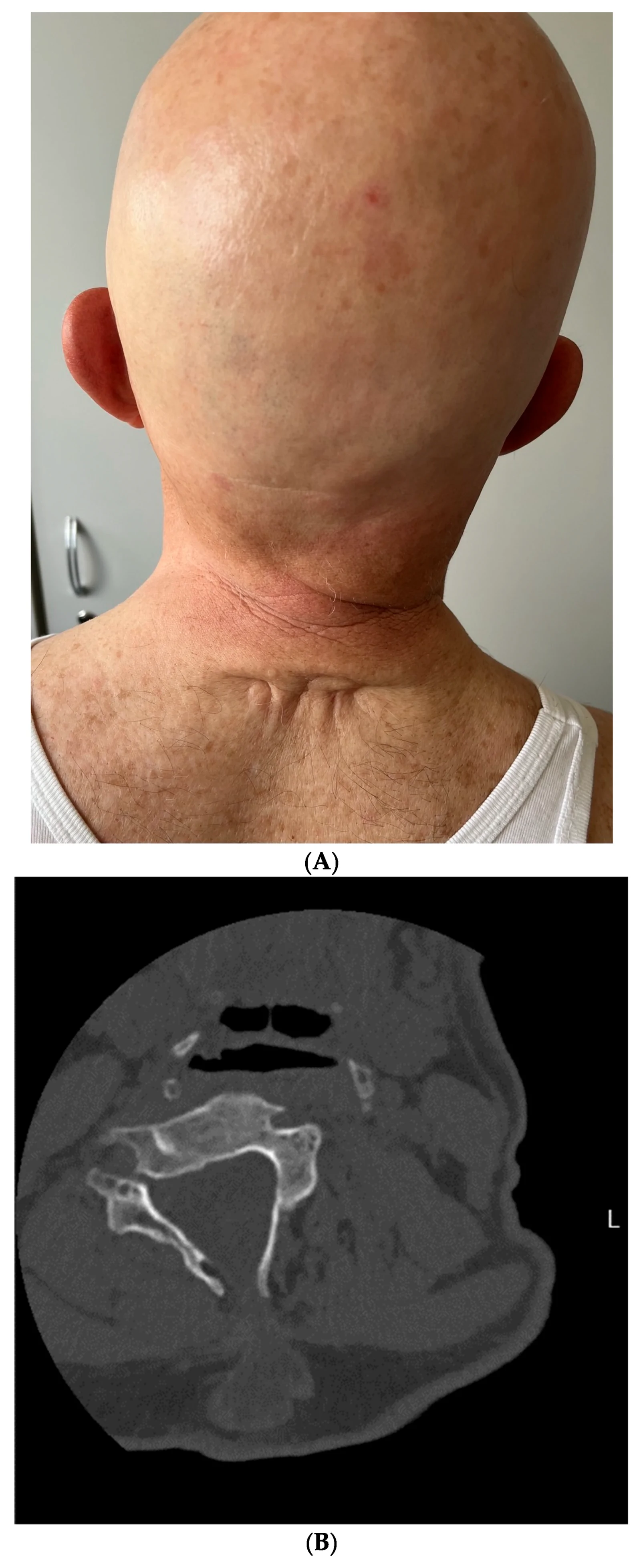

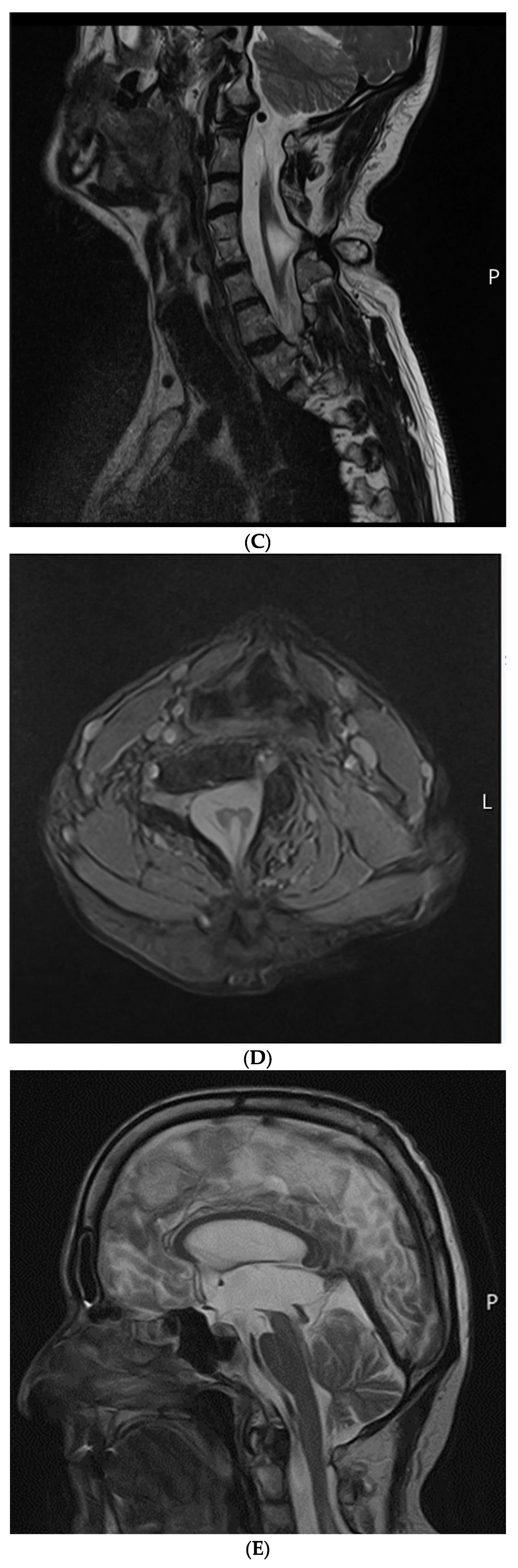
Clinical and radiological features of the illustrative case (**A**–**E**). (**A**) Clinical photograph of the posterior cervical region showing a closed posterior cervical midline cutaneous lesion. A horizontal scar is visible above the C7 prominence, lying flush with the surrounding skin, without evidence of a dermal sinus, local inflammation, swelling, or active CSF leakage. (**B**) Axial CT image at the C4–C5 level demonstrating posterior element non-fusion with absence of the spinous process. (**C**) Sagittal T2-weighted cervical MRI demonstrating congenital C4–C5 block vertebra and a posterior dysraphic osseous defect, with dorsal herniation of the dural sac and neural elements into a subcutaneous sac-like lesion measuring approximately 23 × 31 mm. The findings are most consistent with cervical MMC on radiological grounds. Mild prominence of the central ependymal canal is present, without syringohydromyelia. (**D**) Axial T2-weighted MRI at the C4–C5 level demonstrating a posterior midline subcutaneous cystic lesion at the dysraphic level. Two hemicords are identified, consistent with split cord malformation (SCM), with dorsal extension of neural tissue through the posterior osseous defect into the subcutaneous cystic sac. Associated dorsal tenting of the spinal cord and mild dilatation of the central canal are also noted. (**E**) Sagittal T2-weighted MRI images show ventriculomegaly, interpreted in the context of asymptomatic arrested hydrocephalus. Abbreviations: CSF, cerebrospinal fluid; CT, computed tomography; L, left; MMC, myelomeningocele; MRI, magnetic resonance imaging; P, posterior.

A 62-year-old man presented to a neurosurgeon for the first time in his life on 20 April 2026 in the outpatient clinic due to intermittent left-hand numbness during prolonged driving. The patient reported a congenital posterior cervical midline lesion that had been present since childhood. There was no history of persistent neck pain, progressive upper- or lower-limb weakness, gait disturbance, sphincter dysfunction, CSF leakage, local infection, or other symptoms suggestive of cervical myelopathy. On clinical examination, the patient was pain-free and neurologically intact, defined here as no objective deficit on neurological examination; the intermittent left-hand numbness was a symptom without an examination correlate. Local inspection of the posterior cervical region showed a closed midline cutaneous scar corresponding to the radiological level of the dysraphic lesion (Figure 2A). There was no visible swelling, active CSF leakage, local erythema, tenderness, or evidence of infection.

CT of the cervical spine (September 2025; Figure 2B) demonstrated C4–C5 bony fusion, a posterior element defect, and a retrospinal fluid-density lesion communicating with the spinal canal. MRI of the cervical spine was performed in December 2025 and reviewed by a neuroradiologist (D.B.), demonstrating marked dextroconvex cervical scoliosis with congenital block fusion of the C4–C5 vertebral bodies and posterior elements. At the same level, a midline posterior dysraphic defect allowed dorsal herniation of the dural sac, measuring approximately 23 × 31 mm, containing neural tissue continuous with the spinal cord, most consistent with cervical MMC on radiological grounds; no surgical or histopathological confirmation was obtained, given the conservative management approach (Figure 2C,D). On MRI, the spinal cord appeared dorsally tethered and displaced at this level; tethering was not confirmed surgically. Immediately caudal to the defect, imaging demonstrated an associated SCM consistent with diastematomyelia. No syringohydromyelia was identified, although mild prominence of the central ependymal canal was noted. A supernumerary right C7 cervical rib was also present. Additional workup included MRI of the thoracic spine, lumbar spine, and brain, EMNG of the upper and lower extremities, and somatosensory evoked potentials (motor evoked potentials were not feasible due to technical limitations). EMNG demonstrated mildly reduced conduction velocities along the n. medianus bilaterally, consistent with mild axonal neuropathy on the right and early demyelination of the right n. peroneus; somatosensory evoked potentials were within normal limits. MRI of the thoracic and lumbar spine demonstrated degenerative changes with mild thoracolumbar scoliosis and disc bulging at L1–L2; the conus medullaris terminated at the T12–L1 level, with no evidence of additional dysraphic lesions. Brain MRI revealed mild dilatation of the ventricular system predominantly involving the frontal horns, which was interpreted as possible arrested hydrocephalus in the absence of symptoms or signs of raised intracranial pressure (Figure 2E), and multiple bilateral parasagittal calcifications along the falx cerebri as incidental findings.

The lesion was classified as MMC rather than MC because the sac contained neural tissue continuous with the spinal cord (Figure 2C,D), whereas an MC contains only CSF and meninges. MyCC was not favoured because the central canal was only mildly prominent and there was no cystic terminal dilatation herniating into a meningocele. Without operative or histopathological confirmation, these distinctions remain radiological.

He had never undergone surgical repair, puncture, aspiration, or any previous treatment of the lesion. He also denied any history of meningitis or local inflammatory complications. Family history was non-contributory: no similar congenital abnormalities were known in his parents, and his brother, who died at a young age, had no known comparable condition.

Repeat neurological examination confirmed muscle strength was 5/5 bilaterally in both upper and lower extremities (Medical Research Council scale), with normal tone and trophicity. Deep tendon reflexes were symmetrical; Babinski, Hoffmann, and Tinel signs were negative. Sensory examination, including vibration and proprioception, was intact. Gait and coordination were unimpaired, and sphincter function was preserved.

In the absence of neurological deficits, myelopathic progression, CSF leakage, or local complications, observation and prophylactic untethering were discussed with the patient, including the risks reported in previous cases. The patient declined surgery, and clinical and radiological observation was chosen. At three-month follow-up, the patient remained neurologically intact, with no new deficits or symptom progression. Conservative management with planned clinical and radiological follow-up was continued.

### 3.2. Findings of the Systematic Review

#### 3.2.1. Study Selection

The three databases yielded 1650 records (PubMed 449, Scopus 870, Web of Science 331). After removal of 872 duplicates, 778 records were screened by title and abstract, and 729 were excluded because they concerned anterior cervical pathology, exclusively paediatric populations or lesions outside the cervical spine. Full texts were sought for 49 reports. Six could not be retrieved, and 43 reports were assessed in full. One further report, identified through citation searching, was also assessed, which gave 44 reports in total. Twenty-five were excluded (Figure 1; Supplementary Table S2): 14 because the patients were paediatric (younger than 18 years; criterion E4), seven because the primary diagnosis was not a cervical MC, MMC or MyCC or because the lesion was not in the cervical spine (criterion E1), and four because they were letters, replies or commentaries without patient data (criterion E2). No report was excluded under criteria E3 or E5. Nineteen studies were included (Figure 1). The six reports that could not be retrieved were published between 1949 and 1999. Their titles refer to cervical meningocele, myelomeningocele, meningomyelocystocele or closed cervical dysraphism. Without the full text, it is not known whether they described adults, so their possible contribution cannot be quantified.

The 19 included studies were 18 case reports and one case series, published between 1953 and 2024 in eight countries (Türkiye, seven studies; India, three; USA, three; Iran, two; and Canada, China, Italy and the UK, one each; Table 2). Three of the 18 case reports described several patients, of whom only one in each report was eligible and extracted (Supplementary Table S2). These three reports are counted among the included studies, and their ineligible patients are not counted among the 25 exclusions. The included studies described 23 previously reported adults and, with the present case, 24 patients (Table 2; Supplementary Tables S5 and S6). Scopus and the broadened criterion I4 (Amendment 2, Section 2.2.5) together added six of the 19 studies relative to the earlier search and eligibility set, and a seventh was identified through citation searching (Table 2).

#### 3.2.2. Quality Assessment

The item-level JBI ratings for the 18 case reports are shown in Table 3. Demographic characteristics (18/18), clinical condition at presentation (17/18), diagnostic assessment and results (17/18) and takeaway lessons (16/18) were rated Yes in nearly all reports. The items rated Yes least often were the reporting of adverse or unanticipated events (7 of 16 applicable reports; unclear in seven and not described in two), the history presented as a timeline (12/18; not met in two and unclear in four), the description of the intervention (12 of 16 applicable reports) and the description of the post-intervention condition (13 of 16 applicable reports). Two reports were rated not applicable on these last three items because they stated that no intervention was given [22,30]. Two further reports were rated No on the same three items because no intervention was described [25,26]. In the case series [8], four of ten items were met (condition measured in a standard way, valid identification, reporting of demographics and reporting of outcomes), five were unclear (inclusion criteria, consecutive inclusion, complete inclusion, reporting of clinical information and reporting of site) and one was not applicable (statistical analysis). These ratings describe how completely each report was written. They do not measure risk of bias, and they do not indicate how reliably the reports support conclusions about prognosis or treatment effect (Supplementary Tables S3 and S4).

#### 3.2.3. Characteristics, Management and Outcomes of the Previously Reported Cases

Lesions were grouped by the label used by the original authors: of the 23 previously reported patients, 16 were labelled MC (two rudimentary), one non-terminal MyCC and six MMC (two recurrent or retethered) (Table 4; Supplementary Table S5). The median age was 24 years (range 18–64), and 19 of 23 patients were male. A vertebral level was specified in 20 patients, and it was C4–C5 in five and between C2 and C7 in the others. The remaining three were described as upper cervical (one), suboccipital (one) or without a level (one).

An objective neurological abnormality was documented in 14 of the 21 patients whose examination was described (MC 8/15, MyCC 1/1, MMC 5/5), and the examination was not described in two. In one of the 14, mirror movements were the only abnormal finding. Pain was reported in nine patients (MC 6/16, MMC 3/6). CSF leakage was reported in three, and a cosmetic or asymptomatic lesion was the only reason for presentation in three. Presenting features were not mutually exclusive. Other presentations were complex partial seizures in one patient, mirror movements in two, and behavioural change with headache, vomiting and drowsiness in one [22], in whom autopsy showed a bronchial carcinoma with multiple cerebral metastases.

All six patients aged 40 years or older (42–64 years) had an objective neurological abnormality and were operated on. Among the 12 patients aged 25 years or younger with a documented examination, six had no abnormality. Of the four patients aged 26–39 years, two had an abnormality, one had none and one had no description.

Some vertebral anomaly was reported in 19 of the 23 previously reported patients (Supplementary Table S5). A defect of the posterior arch, laminae or spinous process (including descriptions as a fusion defect or spina bifida) was described in 17 (MC 11/16, MyCC 1/1, MMC 5/6). Two further patients had anomalies of the vertebral bodies alone (small C7 and T1 bodies in one, vertebral wedging in one). No vertebral anomaly was described in four. Segmentation anomalies were described in three (Klippel–Feil syndrome in one). An SCM or related cord split was described in four (one each of type 2 SCM, diplomyelia, limited cord split and lumbar diastematomyelia). A syrinx was reported present in six patients, absent in two and not reported in 15. Dilatation or enlargement of the central canal without a reported syrinx was described in two further patients. Chiari malformation or tonsillar displacement was described in three, hydrocephalus or ventricular dilatation in two, kyphoscoliosis in one and bilateral subependymal nodular heterotopia in one.

Surgery was reported in 19 of the 23 previously reported patients (MC 13/16, MyCC 1/1, MMC 5/6). Of the remaining four, one was untreated (the autopsy case), one declined surgery, and two had no management data, and no follow-up outcome was reported for any of them. The indication for surgery in the 19 operated patients (Supplementary Table S6) was a neurological deficit, progressive symptoms or symptoms attributed to tethering in 12; pain, CSF leakage or cyst resection without progressive deficit in four; and cosmetic reasons in three. Operative techniques were heterogeneous, but release of tethering structures (bands, attachments, stalk, fibrous tract or roots) was part of all 18 operations for which operative details were reported. Operative details were not reported in one patient.

Among the 19 operated patients, the outcome was IMP in 10, INT in five, STB in two, and could not be classified in two (no postoperative neurological status was reported in either). Thirteen operated patients had an objective neurological abnormality before surgery (one of them was operated on for pain and cyst resection). Of these, 10 improved, two remained stable, and the outcome was not reported in one. The five patients classified as intact were intact before surgery. None of the 17 operated patients with a classified outcome was reported to be neurologically worse at last follow-up. Outcome was mostly described in words. A graded measure was reported both before and after surgery in one patient (Pang grade 3 to 2), and before surgery only in two (a visual analogue scale in one, the Nurick grade in one).

Adverse events were reported in two operated patients: a transient fall of motor evoked potentials to 10% of baseline on neck flexion, resolving within 5 min without added deficit, and faecal incontinence during circumferential dissection, attributed to cord stretching, with an uneventful postoperative course. Adverse events were explicitly absent in 10 (five of them from a series-level statement) and not reported in seven. Recurrence or progression was documented as absent in seven and not reported in 12. Follow-up was reported for 15 of the 19 operated patients (median 12 months, range 1–24). It was 12 months or longer in 10 and shorter than 12 months in five (1 to 6 months), and it was not reported in the other four.

Four patients (aged 18, 35, 43 and 47 years) presented with progressive neurological symptoms after earlier surgery on the same lesion. All four were reoperated and were classified as IMP. Follow-up was 6, 4 and 1 months in three of them and was not reported in one.

Histopathology was reported for 11 of the 19 operated lesions. In MC (seven reports), meningothelial cells with psammoma bodies were recorded in four. The absence of neural elements was stated in six, and neural tissue was not commented on in one. Dysplastic glia and neurons within the tethering band were described in one. The MyCC contained glial tissue with adipose tissue. In MMC (three reports), histology showed meningothelial cells with psammoma bodies and glial tissue in one, neuroglial tissue within fibrosis in one, and a pleomorphic leiomyosarcoma with tumour-free margins in one, in a lesion labelled by the original authors as a cervical MMC [29]. That patient, aged 18 years, also received radiotherapy and was reported as asymptomatic at 12 months.

#### 3.2.4. Present Case in Relation to the Published Cases

The present patient (62 years) is the second oldest of the 24 patients and the only patient aged 40 years or older without a documented neurological abnormality. None of the 23 previously reported patients was described with the combination of five anomalies seen in the present patient (segmentation anomaly, SCM, scoliosis, supernumerary rib and hydrocephalus). No previously reported patient had more than two of these features, and two were present in three patients (segmentation anomaly combined with SCM, with arrested hydrocephalus or with kyphoscoliosis; one patient each). A supernumerary rib was not described in any previously reported patient. The present patient is one of three who were not operated on, the other two being the untreated autopsy case and a patient who declined surgery and had no reported outcome. He is the only non-operated patient with a reported follow-up (3 months, with no new deficit or symptom progression).

## 4. Discussion

To our knowledge, this is the first systematic synthesis of adult posterior cervical spinal dysraphism, identifying 23 previously reported adult patients with posterior cervical cystic spinal dysraphism in 19 studies and describes one further patient managed by observation, who is considered separately throughout. Most reported patients were male (19 of 23), the median age was 24 years (range 18 to 64), and lesions were labelled MC in 16 patients (two rudimentary), MMC in six and MyCC in one. The clinical spectrum was heterogeneous, ranging from a cosmetic or asymptomatic lesion to pain, CSF leakage, an objective neurological abnormality and symptomatic recurrence after earlier surgery.

The present case, which had no objective neurological deficit at presentation and was managed by observation, adds to this spectrum. It contrasts with most published adult posterior cases, in which surgical excision and untethering were performed. Among the identified cases, the only older patient was the 64-year-old reported by Bozkurt et al., who presented with neuropathic pain, neurological deficits, and syringomyelia [1]. Although this case does not establish a benign natural history or the safety of observation, it illustrates that extensive structural anomalies may be present in an adult without an objective neurological deficit and without a syrinx. His earlier neurological status is unknown, and follow-up is limited to 3 months. After an informed discussion of the reported cases and available management options, the patient declined surgery, and structured clinical follow-up with MRI of the brain and cervical spine every 6 months was planned.

### 4.1. Terminological, Embryology and Radiological Spectrum

Adult posterior cervical spinal dysraphism is terminologically heterogeneous, with cases described as MC, MMC, rudimentary MC, retethered MMC, and non-terminal MyCC. The classification of cystic cervical lesions has long been disputed. Habibi et al. considered that all of them contain neural tissue and should therefore be called MMC [12], whereas Akay et al. labelled their cervical lesion a meningocele, reasoning that the lesions contained no spinal cord [20]. Pang et al. defined limited dorsal myeloschisis (LDM) by a focal closed midline defect and a fibroneural stalk linking the skin lesion to the cord, with saccular and non-saccular forms [6], and cervical saccular LDM has since been proposed as the term for lesions previously called cervical MMC [14]. Another classification of cystic dysraphism is based on the content of the cyst and distinguishes MMC, MyCC and MC [3]. Wang et al., on the basis of a single adult case, argued that rudimentary MC, which has been referred to as a cutaneous meningioma, is a remnant of a neural tube defect and belongs in the classification of cervical spinal dysraphism [27]. In this review, lesions were grouped by the label used in the original report and are presented separately by group, without implying biological equivalence.

The embryological interpretation is also provisional. Incomplete disjunction of cutaneous and neural ectoderm has been proposed for LDM [6], and it has been suggested that true MMC results from failure of primary neurulation whereas cervical saccular LDM results from a delayed abnormality in the final stages of neurulation [14]. The origin of LDM itself is still debated [32], and the sequence of human neural tube closure remains controversial [7]. These mechanisms are therefore proposals, not established consensus, and the present data do not test them.

Histopathology was reported for 11 of 19 operated lesions. Meningothelial cells with psammoma bodies were described in four of seven MC reports, and glial or neuroglial tissue in the single MyCC and in two of three MMC reports. A pleomorphic leiomyosarcoma with tumour-free resection margins was reported in an 18-year-old patient with a chronically neglected lesion labelled cervical MMC [29]. This single report documents what its authors describe as a rare complication of a neglected lesion, but it does not establish an increased or quantifiable risk of malignancy, and the lesion label rests on the original authors’ description, who also categorised the tumour as arising in an MC.

The diagnosis in the present patient is radiological. It rests on CT and MRI and on neurological examination and electromyoneurography (EMNG), and there is no operative or histopathological confirmation. A saccular LDM remains in the differential diagnosis, because a short stalk may not be visible on sagittal and axial heavily T2-weighted images alone, and a dorsally bent cord extending into the sac has been described in saccular LDM when the stalk tethers strongly [33]. Therefore, the lesion is best regarded as a radiologically suspected MMC, and the present case carries limited weight in the synthesis.

### 4.2. Presentation, Age and Associated Anomalies

An objective neurological abnormality was documented in 14 of 21 patients with a described examination. Because asymptomatic adults are unlikely to be reported, these data cannot show how often adults with such lesions develop deficits. A syrinx was reported present in six patients, absent in two and not reported in 15, so no prevalence can be given.

All six patients aged 40 years or older (42 to 64 years) had an objective neurological abnormality and were operated on, whereas six of 12 patients aged 25 years or younger with a documented examination had none. This pattern is compatible with progression with chronicity, as suggested by a positive correlation between neurological grade and age [6] and by older age at surgery as a predictor of deficit in pooled paediatric data [14]. It can equally reflect selective reporting of symptomatic older adults, so it generates a hypothesis and does not describe natural history.

Several mechanisms have been proposed to explain late neurological deterioration. Konya et al. [9] emphasised that cervical cord tethering acts predominantly dorsally or horizontally. Duz et al. [8] proposed that repetitive cervical flexion–extension movements may produce cumulative microtrauma. Laurent et al. [11] noted that repeated movement in a fixed cord may lead to ischemic injury over time. All of them remain unproven for adult posterior cervical dysraphism.

Vertebral anomalies, including defects of the posterior arch, segmentation anomalies and split cord malformation, were frequent among the previously reported patients (19 of 23). The present patient had five findings (segmentation anomaly with C4–C5 block vertebra, SCM, scoliosis, supernumerary right C7 rib and ventriculomegaly interpreted as possible arrested hydrocephalus), and no previous patient had more than two of them. All five rest on imaging, and the hydrocephalus is an interpretation of ventriculomegaly, not a confirmed diagnosis. Hydrocephalus and Chiari malformation were reported less often in the published adult patients than in a paediatric series of cervical saccular LDM (39.47% and 28.95%, respectively) [14], which may reflect survival or lesion type.

Whether these anomalies share a developmental origin cannot be determined from the available data. The unified theory of SCM proposes adhesions between ectoderm and endoderm with an accessory neurenteric canal that bisects the notochord and gives rise to two hemineural plates [34], and an exceptionally rare form of LDM involving the dorsal surface of one hemicord in a type II SCM has been reported [35]. A congenital defect in the formation or segmentation of the cervical spine defines Klippel–Feil syndrome, a heterogeneous condition that may be associated with abnormalities of other organ systems [36]. These reports show that coexistence is recognised, but they do not demonstrate a common mechanism for the combination seen here, and no causal link between the associated anomalies and the absence of an objective deficit in this patient should be inferred. Their relevance is mainly practical. SCM has been associated with poorer outcomes after untethering in adult-onset tethered cord syndrome [37]. Therefore, the SCM is the associated finding that would matter for planning any future operation. None of the other findings led to additional measures beyond the planned MRI follow-up.

The present patient reported intermittent left hand numbness during prolonged driving, without an examination correlate. EMNG showed mildly reduced median nerve conduction velocities bilaterally and early demyelination of the right peroneal nerve, and somatosensory evoked potentials were normal. These are peripheral findings, and their relationship to the cervical lesion cannot be determined, so the patient is described as having no objective neurological deficit at 62 years.

### 4.3. Management Decision Making

Surgery was reported in 19 of 23 previously reported patients, and the main indication was most often a neurological deficit, progressive symptoms or symptoms attributed to tethering. Operative techniques were heterogeneous, but the release of tethering structures formed part of all 18 operations with reported operative details, in keeping with reports that excision of the sac without intradural untethering is insufficient [3].

Among 17 operated patients with a classified outcome, none were reported to be neurologically worse at last follow-up. Follow-up was short (median 12 months, range 1 to 24), and adverse events and recurrence were incompletely reported. These frequencies describe published cases. They are not estimates of the effectiveness or safety of surgery, and without a comparison group the favourable reports cannot be read as superiority over observation.

The proportion of operated patients (19 of 23) should not be read as the proportion of adults who need surgery. Patients with progressive symptoms are more likely to be treated and to be published, which combines confounding by indication with publication bias. The narrowing of criterion I4 in amendment 1 may have favoured the exclusion of conservatively managed and incidentally identified cases, and its widening in amendment 2 reverses this, but the published record itself remains skewed toward symptomatic and operated patients.

Four patients presented with progressive neurological symptoms after earlier surgery on the same lesion. All four were reoperated and classified as improved, but follow-up was short (1 to 6 months where reported). In infants with cervical MMC, five of six children treated by sac resection without release of the internal tethering structures later deteriorated and improved after re-exploration with release [38], while in adults with tethered cord syndrome and an associated lipoma or dysraphic cyst, secondary operations were associated with limited long-term benefit [39]. The reoperated patients therefore support long-term follow-up, but the data cannot show durable benefit.

The present patient is the only non-operated patient with a reported follow-up, during which there was no new deficit or symptom progression. The other two non-operated patients were an untreated autopsy case and a patient who declined surgery and had no reported outcome. Observation was chosen in the present case because there was no objective deficit, myelopathic progression, CSF leakage or local complication. No conclusion on the safety of observation or on natural history can be drawn from this, and information on the natural course of such lesions in adults is lacking [1]. Recommendations in the original reports are divided and rest on opinion. Several authors advised early surgery once the diagnosis is made [8,9,10], whereas others consider close follow-up reasonable in asymptomatic adults after informed consent, acknowledging that untethering is not risk-free [3,27].

Evidence from other tethering lesions cannot be transferred directly. In adults with tethered cord syndrome, a conservative approach was considered warranted in patients without neurological deficits, although the clinical recurrence rate in conservatively treated patients was 21% at 10 years [39]. Observation can therefore be regarded only as a hypothesis-generating option for individualised decision making and not as a general recommendation, and it requires informed consent, baseline and follow-up imaging, and longitudinal neurological assessment. To our knowledge, no guideline specific to the surveillance of unrepaired posterior cervical dysraphism in adults exists. The Spina Bifida Association guidelines, which address mainly open myelomeningocele, advise clinical review of adults at 12-month intervals, with imaging used judiciously according to clinical judgement, and they rest largely on clinical consensus [40]. The follow-up planned for the present patient, with MRI of the brain and cervical spine every 6 months, is more intensive than this and was set individually, not on the basis of a guideline.

### 4.4. Limitations and Future Directions

Several limitations should be acknowledged. The main limitation is the nature of the evidence. All included studies were case reports or a single case series, which precluded meta-analysis. Publication bias is likely, as symptomatic and surgically treated cases are more readily reported. No denominators exist, so prevalence, the proportion of patients who need surgery and natural history cannot be estimated, and confounding by indication cannot be excluded. Given the absence of a comparator group, the favourable outcomes reported after surgery cannot be interpreted as evidence of comparative effectiveness or safety relative to observation.

Terminology and diagnostic criteria were heterogeneous, and cases were grouped by the original authors’ labels without reclassification. Some lesions labelled MC or MMC may therefore meet current criteria for LDM, and the pooled figures should not be read as describing a homogeneous disease. Reporting was incomplete, particularly for syrinx status, adverse events, neurological grading, follow-up, and outcome. Follow-up was generally short, which precludes conclusions regarding delayed deterioration, retethering, malignant transformation or the long-term safety of observation. The JBI checklists describe how completely each report was written, not the certainty of the evidence.

Search limitations also apply. Embase was not searched, because of a lack of institutional access. Six reports could not be retrieved, and it is not known whether they described adults. The protocol was amended twice after the initial searches and with knowledge of the earlier results (Section 2.2.5), which could have changed the number and characteristics of the included studies.

The present case rests on a radiological diagnosis without operative or histopathological confirmation and has 3 months of follow-up. It is the only conservatively managed patient with reported follow-up, so any suggestion that observation is an option is hypothesis-generating and rests on expert opinion rather than comparative evidence.

Future research should prioritise multicentre registries standardising lesion classification, MRI/CT descriptors, neurological grading, histopathology and long-term follow-up, so that surgical and conservative management can eventually be compared reliably.

## 5. Conclusions

Adult posterior cervical cystic spinal dysraphism is rarely reported, and the available evidence is scarce, heterogeneous and limited to case reports and a single small series. This review characterises the reported adult cases and one additional radiologically diagnosed case, which differ in terminology, presentation and management, and it highlights major gaps in current knowledge. Published cases were predominantly symptomatic and surgically treated, and operated patients with a classified outcome were described as improved, intact or stable. These descriptive findings are subject to publication bias, confounding by indication and short follow-up, and therefore do not establish the effectiveness or safety of surgery. The available evidence is insufficient to define the natural history, optimal management or long-term outcomes, or to compare surgery with observation. The present case is hypothesis-generating only. Treatment decisions in adults should be individualised according to clinical status and imaging findings, with informed consent and longitudinal follow-up. Addressing these gaps requires standardised multicentre data collection with uniform terminology, explicit diagnostic criteria and long-term follow-up.

## Figures and Tables

**Table 1:** Eligibility criteria for study inclusion and exclusion.

Code	Criteria
**Inclusion Criteria**	
I1	Case reports, case series, cohort studies, or observational studies reporting cervical MC, MMC, or MyCC in patients aged ≥18 years
I2	Diagnosis confirmed by MRI, CT, surgical exploration, or autopsy
I3	Lesion located at the cervical level (C1–C7); mixed-level studies eligible if cervical cases were reported separately
I4	Sufficient demographic and clinical detail for case-level extraction (age, sex, and neurological status), regardless of treatment or outcome reporting
**Exclusion Criteria**	
E1	Lesions located exclusively at the thoracic, lumbar, or sacral level or extending from the cervical into the thoracic spine (cervicothoracic lesions); anterior or anterolateral cervical location
E2	Editorials, letters, opinion pieces, or review articles without original patient data
E3	Insufficient demographic and clinical detail for case-level extraction
E4	Exclusively paediatric population (all patients younger than 18 years)
E5	Studies reporting exclusively genetic, embryological, or molecular findings without a clinically described patient phenotype

Abbreviations: MC, meningocele; MMC, myelomeningocele; MyCC, myelocystocele; MRI, magnetic resonance imaging; CT, computed tomography.

**Table 2:** Reported adult posterior cervical spinal dysraphism cases, with the present case.

Study (Year), Country	Age/Sex	Lesion Type	Level	Prior Repair	Syrinx	Presenting Symptoms	Associated Anomalies	Management	Histopathology	Follow-Up; Outcome
Meningocele (MC), *n* = 16										
Bozkurt et al., 2024, Türkiye [1]	64/M	MC	C5–6	No	Yes	Neuropathic pain, numbness and weakness; congenital neck lesion	Posterior arch fusion defect	Excision; untethering	No neural tissue; meningothelial cells, psammoma bodies	12 mo (also 6); improved (minimum symptoms); residual left-arm tingling
Duz et al., 2008, Türkiye (Pt 1) [8]	22/M	MC	C2	No	NR	Congenital midline mass; intermittent CSF leak; no pain reported	C2 fusion defect	Excision; untethering; dural repair	Meningothelial heterotopia; psammoma bodies; no neural elements	12 mo; neurologically intact; no recurrence; tethering resolved
Duz et al., 2008, Türkiye (Pt 2) [8]	22/M	MC	C4–5	NR	NR	Neck pain; posterior cervical mass	Cervical fusion defect	Cyst excision; untethering	NR	12 mo; neurologically intact; no neurological disturbance
Duz et al., 2008, Türkiye (Pt 3) [8]	20/M	MC	C3–4	NR	NR	Neck pain; posterior mass; left finger weakness	C3–4 fusion defect	Excision; untethering	No neural/neoplastic components	12 mo; finger weakness resolved; no recurrence
Duz et al., 2008, Türkiye (Pt 4) [8]	20/M	MC	C6–7	NR	NR	Neck pain; posterior cervical mass	Small C7–T1 vertebral bodies	Excision; untethering	NR	12 mo; neurologically intact; no neurological disturbance
Duz et al., 2008, Türkiye (Pt 5) [8]	18/M	MC	C6–7	NR	NR	Neck pain; cervical mass; hypoactive biceps/triceps reflexes	Fusion defects; SCM type 2; Klippel–Feil	Excision; release of attachments; duraplasty	No neural/neoplastic components	24 mo; no recurrence or neurological deterioration
Konya et al., 2006, Türkiye [9]	47/M	MC	C4–5	No	NR	Leakage of colourless fluid (3 mo) from ulcerated sac; gait difficulty; leg stiffness	Tethered cord; laminar dysplasia	Excision; untethering; duraplasty	No neural/neoplastic tissue	12 mo; walking difficulty and leg stiffness resolved; no recurrence
Akay et al., 2003, Türkiye [20]	21/M	MC	C2–3	No	No	Asymptomatic cervical lesion; cosmetic indication	C2–3 spina bifida; cord tethering	Excision; untethering	NR	24 mo; neurologically intact; intraoperative faecal incontinence during dissection (duration not stated; attributed to cord stretching)
Shamji & Ibrahim, 2015, Canada [21]	22/F	MC	NR	NR	NR	Enlarging congenital cutaneous lesion; bilateral Hoffmann/UMN signs	Dorsal cord tethering by connecting tract	Laminectomy; stalk resection; untethering; duraplasty	NR	NR; no visible recurrence or neurological progression
Hepburn, 1953, UK [22]	25/M	Presumed MC	C2	NR	NR	Behavioural change; head/neck pain; transient suboccipital swelling	Bifid hypertrophied C2; meninges-lined channel	None	NR (autopsy description only)	Died 10 days after admission; autopsy: bronchial carcinoma with multiple cerebral metastases
Erdinçler, 2002, Türkiye (Case 2) [23]	18/F	MC, repaired	C2–3	Yes	NR	Progressive gait disturbance; UMN signs; mirror movements	Cerebellar tonsil displacement to C2; mild ventricular dilatation; spina bifida; central canal dilatation	Posterior fossa craniectomy; C1–2 laminectomy; untethering; duraplasty	NR	NR; gait improved; mirror movements persisted
Wu & Scott, 1990, USA (Case 1) [24]	43/M	Probable MC	C3–4	Yes	Yes	Progressive loss of hand fine motor skills; weakness and sensory deficits	Fibrous tract through C3–4 to dura	C2–5 laminectomy; aberrant root resection; syrinx drainage	NR	6 mo; improved; near-normal strength with residual left hand weakness
Lotfi et al., 2011, Iran [25]	24/M	MC	Upper cervical	NR	NR	Complex partial seizures; congenital neck mass	Bilateral subependymal nodular heterotopia	NR	NR	NR
Odabaşı et al., 1998, Türkiye [26]	20/M	MC	Suboccipital	NR	NR	Mirror movements; suboccipital mass	Undefined anomaly posterior to cord–medulla junction	NR	NR	NR
Rahimizadeh et al., 2023, Iran [3]	34/M	Rudimentary MC	C4–5	No	NR	Congenital solid mass; cosmetic indication	Bifid laminae; limited cord split	Excision; untethering	Meningothelial cells; psammoma bodies	NR; uneventful recovery
Wang et al., 2013, China [27]	21/F	Rudimentary MC	C3	NR	NR	Cosmetic indication	Hair collar sign; neuroglial band; arachnoid cyst; central canal enlargement	Excision; untethering; cyst resection	Meningothelial cells/psammoma bodies; dysplastic glia/neurons	12 mo; no neurological deficit
**Myelocystocele (MyCC), *n* = 1**										
Raheja et al., 2014, India [28]	42/M	Non-terminal MyCC	C6–7	NR	Yes	Progressive spastic quadriparesis, sensory loss and UMN bowel/bladder dysfunction	Mild Chiari II; arrested hydrocephalus; vertebral fusion; open-book cord	Tethering-band excision; adhesiolysis	Glial tissue with adipose tissue; confirms MyCC	6 mo; improved; Pang grade 3→2
**Myelomeningocele (MMC), *n* = 7 (six previously reported cases and the present case)**										
Denaro et al., 2008, Italy [5]	52/M	MMC	C4–5	No	Yes	Progressive bilateral upper-limb weakness/paresthesia; cervical mass	Diplomyelia; spina bifida; syrinx; posterior tethering	Sac resection; stalk excision; untethering	Meningothelial foci/psammoma bodies; glial/astrocytic bands	6 mo; improved; asymptomatic; syrinx shrinking on MRI
Noureldine et al., 2020, USA [10]	47/M	Recurrent MMC	C4–6	Yes	No	Neck pain; loss of finger dexterity; gait instability	Absent C5 spinous process; prior repair scar	Untethering; MMC repositioned into dura; primary closure	NR	1 mo; pain resolved; neurologically stable
Laurent et al., 2020, USA [11]	35/M	Retethered MMC	C4–5	Yes	Yes	Progressive left upper-limb weakness, numbness and hand clumsiness	Posterior element defect; lumbar diastematomyelia	Untethering; MMC repair	Neuroglial/meningeal bands in fibrosis	4 mo; near-complete symptom resolution; full hand strength
Mohindra et al., 2021, India [29]	18/M	MMC with malignant transformation	C3	No^b^	NR	Congenital swelling; spastic paraparesis; CSF leak/ulceration	C3 bony defect; cord tethering	Excision; untethering; radiotherapy	Pleomorphic leiomyosarcoma; tumour-free margins	12 mo; asymptomatic; MRI at 9 mo showed untethered cord
Aribal et al., 2016, Türkiye [30]	28/F	MMC	C6	NR	Yes	Chronic cervical/dorsal pain and sensory loss	Multiple fusion defects; Chiari; kyphoscoliosis; syringohydromyelia	Surgery declined; conservative follow-up	N/A	NR; no outcome reported
Chitale & Deshmukh, 2021, India [31]	31/M	MMC	C5–7	NR	NR	Cervical/upper-limb pain and numbness; fatty cervical lump	Vertebral body wedging	MMC correction; (operative details not reported)	NR	NR; no postoperative complications
***Present case, Bosnia and Herzegovina***	***62/M***	***MMC (radiologically suspected)***	***C4–5***	***No***	***No***	***Intermittent left-hand numbness; congenital cervical lesion; neurologically intact***	***C4–5 block vertebra/posterior defect; split cord malformation; supernumerary C7 rib; scoliosis; arrested hydrocephalus***	***Observation***	***N/A***	***3 mo; no new neurological deficit***

Note: Cases are grouped by the lesion type given by the original authors (no reclassification). The present case (bold/italic) is the last row. Abbreviations: CSF, cerebrospinal fluid; MC, meningocele; MMC, myelomeningocele; MRI, magnetic resonance imaging; MyCC, myelocystocele; mo, months; N/A, not applicable; NR, not reported; Pt, patient; SCM, split cord malformation; UMN, upper motor neuron.

**Table 3:** JBI item-level ratings, case reports (*n* = 18).

Item	Yes	No	Unclear	N/A
Q1 Demographic characteristics	18	0	0	0
Q2 History presented as a timeline	12	2	4	0
Q3 Clinical condition at presentation	17	0	1	0
Q4 Diagnostic assessment and results	17	0	1	0
Q5 Intervention described	12	2	2	2
Q6 Post-intervention clinical condition	13	2	1	2
Q7 Adverse or unanticipated events	7	2	7	2
Q8 Takeaway lessons	16	0	2	0

Note: The ratings are reported item by item; no summed score or risk-of-bias category is assigned. Abbreviation: N/A, not applicable.

**Table 4:** Characteristics of the 23 previously reported cases by lesion group, and of the present case.

Variable	MC (*n* = 16)	MyCC (*n* = 1)	MMC (*n* = 6)	All Reported Cases (*n* = 23)	Present Case
Age, years, median (range)	22 (18–64)	42	33 (18–52)	24 (18–64)	62
Male sex, n/N	13/16	1/1	5/6	19/23	male
Prior repair of the same lesion, yes/no/NR	2/5/9	0/0/1	2/2/2	4/7/12	no
Syrinx, present/absent/NR	2/1/13	1/0/0	3/1/2	6/2/15	absent
Objective neurological abnormality, yes/no/NR	8/7/1	1/0/0	5/0/1	14/7/2	no
Pain, n/N	6/16	0/1	3/6	9/23	none reported
CSF leakage, n/N	2/16	0/1	1/6	3/23	no
Cosmetic or asymptomatic lesion as the only reason for presentation, n/N	3/16	0/1	0/6	3/23	no
Posterior arch, laminar or spinous defect reported, n/N	11/16	1/1	5/6	17/23	yes
Split cord malformation or cord split (any level), n/N	2/16	0/1	2/6	4/23	yes
Chiari malformation or tonsillar displacement, n/N	1/16	1/1	1/6	3/23	not described
Hydrocephalus or ventricular dilatation, n/N	1/16	1/1	0/6	2/23	yes (arrested)
Management: surgery/no surgery/NR	13/1/2	1/0/0	5/1/0	19/2/2	observation
Outcome among operated: IMP/INT/STB/NR	5/5/2/1 (*n* = 13)	1/0/0/0 (*n* = 1)	4/0/0/1 (*n* = 5)	10/5/2/2 (*n* = 19)	not applicable
Follow-up, months, median (range)	12 (6–24), *n* = 10	6, *n* = 1	5 (1–12), *n* = 4	12 (1–24), *n* = 15	3
Adverse events among operated: reported/none/NR	2/7/4	0/0/1	0/3/2	2/10/7	not applicable
Recurrence or progression among operated: documented absent/NR	6/7	1/0	0/5	7/12	none at 3 months
Histopathology reported among operated, n/N	7/13	1/1	3/5	11/19	not applicable

Abbreviations: CSF, cerebrospinal fluid; IMP, improved; INT, neurologically intact; MC, meningocele (including rudimentary MC); MMC, myelomeningocele; MyCC, myelocystocele; NR, not reported; STB, stable.

## Data Availability

The clinical data presented in the case report were collected during standard patient care and are described within the article. Data for the systematic review were extracted from published literature according to the predefined methodology, and are available within the article and its Supplementary Tables.

## Supplementary Materials

The following supporting information can be downloaded at https://www.mdpi.com/article/10.3390/brainsci16101107/s1, Table S1: Database search strings; Table S2: Full-text exclusion log; Table S3: JBI Critical Appraisal Checklist for Case Reports; Table S4: JBI Critical Appraisal Checklist for Case Series; Table S5: Clinical, radiological, and diagnostic data of included cases; Table S6: Operative management, histopathology, follow-up, and outcome of included cases; PRISMA 2020 Checklist.

## Abbreviations

The following abbreviations are used in this manuscript:

CSF	Cerebrospinal fluid
CT	Computed tomography
EMNG	Electromyoneurography
JBI	Joanna Briggs Institute
LDM	Limited dorsal myeloschisis
MC	Meningocele
MMC	Myelomeningocele
MRI	Magnetic resonance imaging
MyCC	Myelocystocele
NTD	Neural tube defect
PRISMA	Preferred Reporting Items for Systematic Reviews and Meta-Analyses
PROSPERO	International Prospective Register of Systematic Reviews
SCM	Split cord malformation
UMN	Upper motor neuron

## References

[1] Bozkurt I., Durlanik A., Canbay S. (2024). Neglected Case of Cervical Meningocele in an Adult. Turk. Neurosurg..

[2] Meyer-Heim A.D., Klein A., Boltshauser E. (2003). Cervical Myelomeningocele—Follow-up of Five Patients. Eur. J. Paediatr. Neurol..

[3] Rahimizadeh A., Ahmadi S.A., Mohammadi Moghadam A., Rahimizadeh S., Williamson W., Amirzadeh M., Hajaliloo Sami S. (2023). Neglected Cervical Meningocele in an Adult: Illustrative Case. J. Neurosurg. Case Lessons.

[4] MRC Vitamin Study Research Group (1991). Prevention of neural tube defects: Results of the Medical Research Council Vitamin Study. Lancet.

[5] Denaro L., Padoan A., Manara R., Gardiman M., Ciccarino P., d’Avella D. (2008). Cervical Myelomeningocele in Adulthood: Case Report. Neurosurgery.

[6] Pang D., Zovickian J., Oviedo A., Moes G.S. (2010). Limited dorsal myeloschisis: A distinctive clinicopathological entity. Neurosurgery.

[7] Copp A.J., Stanier P., Greene N.D.E. (2013). Neural Tube Defects: Recent Advances, Unsolved Questions, and Controversies. Lancet Neurol..

[8] Duz B., Arslan E., Gönül E. (2008). Cervical Congenital Midline Meningoceles in Adults. Neurosurgery.

[9] Konya D., Dagcinar A., Akakin A., Gercek A., Ozgen S., Pamir M.N. (2006). Cervical Meningocele Causing Symptoms in Adulthood: Case Report and Review of the Literature. J. Spinal Disord. Tech..

[10] Noureldine M.H.A., Peto I., Jallo G.I., Agazzi S. (2020). Symptomatic Recurrence of Cervical Spine Myelomeningocele in an Adult Patient. World Neurosurg..

[11] Laurent D., Bardhi O., Gregory J., Yachnis A., Governale L.S. (2020). Pediatric Pathology All Grown up-An Interesting Case of Adult Tethered Spinal Cord. Surg. Neurol. Int..

[12] Habibi Z., Nejat F., Tajik P., Kazmi S.S., Kajbafzadeh A.M. (2006). Cervical myelomeningocele. Neurosurgery.

[13] Ricci E., Meola A., Scali I., Manganotti P., Tacconi L. (2025). Anterior Cervical Meningocele: Systematic Review of the Literature and Illustrative Case. J. Clin. Med..

[14] Maroufi S.F., Dabbagh Ohadi M.A., Hosseinnejad I., Tayebi Meybodi K., Takzare A., Ashjaei B., Nejat F., Habibi Z. (2024). Cervical Saccular Limited Dorsal Myeloschisis, so-Called “Cervical Myelomeningocele”: Long-Term Follow-up of a Single-Center Series and Systematic Review. J. Neurosurg. Pediatr..

[15] Page M.J., McKenzie J.E., Bossuyt P.M., Boutron I., Hoffmann T.C., Mulrow C.D., Shamseer L., Tetzlaff J.M., Akl E.A., Brennan S.E. (2021). The PRISMA 2020 Statement: An Updated Guideline for Reporting Systematic Reviews. BMJ.

[16] Hair K., Bahor Z., Macleod M., Liao J., Sena E.S. (2023). The Automated Systematic Search Deduplicator (ASySD): A Rapid, Open-Source, Interoperable Tool to Remove Duplicate Citations in Biomedical Systematic Reviews. BMC Biol..

[17] Ouzzani M., Hammady H., Fedorowicz Z., Elmagarmid A. (2016). Rayyan-a Web and Mobile App for Systematic Reviews. Syst. Rev..

[18] Aromataris E., Lockwood C., Porritt K., Pilla B., Jordan Z. (2024). JBI Manual for Evidence Synthesis.

[19] Munn Z., Barker T.H., Moola S., Tufanaru C., Stern C., McArthur A., Stephenson M., Aromataris E. (2020). Methodological Quality of Case Series Studies: An Introduction to the JBI Critical Appraisal Tool. JBI Evid. Synth..

[20] Akay K.M., Gönül E., Ocal E., Timurkaynak E. (2003). The Initial Treatment of Meningocele and Myelomeningocele Lesions in Adulthood: Experiences with Seven Patients. Neurosurg. Rev..

[21] Shamji M.F., Ibrahim A. (2015). Cervical Meningocele. N. Engl. J. Med..

[22] Hepburn W.D. (1953). Multiple cerebral metastases without papilloedema: Cervical meningocoele as a possible decompressing mechanism. Confin. Neurol..

[23] Erdinçler P. (2002). Cervical cord tethering and congenital mirror movements: Is it an association rather than a coincidence?. Br. J. Neurosurg..

[24] Wu J.K., Scott R.M. (1990). Myelopathy presenting decades after surgery for congenital cervical cutaneous lesions. Neurosurgery.

[25] Lotfi M., Iranpour P., As’adi K. (2011). Cervical meningocele associated with subependymal nodular heterotopia. Clin. Imaging.

[26] Odabaşi Z., Gökçil Z., Kütükçü Y., Vural O., Yardim M. (1998). Mirror movements associated with cervical meningocele: Case report. Minim. Invasive Neurosurg..

[27] Wang H., Yu W., Zhang Z., Lu Y., Li X. (2013). Cervical Rudimentary Meningocele in Adulthood. J. Neurosurg. Spine..

[28] Raheja A., Gupta D.K., Nalwa A., Suri V., Sharma B.S. (2014). Non-Terminal Cervical Myelocystocele: Unusual Cause of Spastic Quadriparesis in an Adult. Neurol. India.

[29] Mohindra S., Batish A., Tripathi M., Nambiyar K., Gupta K. (2021). Leiomyosarcoma in a Cervical Myelomeningocele: A Rare Complication in a Neglected Case. Childs Nerv. Syst..

[30] Aribal S., Zorlu E., Ozyurek S. (2016). Cervical myelomeningocele associated with multiple spinal anomalies. Spine J..

[31] Chitale N.V., Deshmukh M.K. (2021). Tethering of the spinal cord in cervical region in adult male patient. Pan Afr. Med. J..

[32] Kim J.W., Wang K.-C., Chong S., Kim S.-K., Lee J.Y. (2020). Limited dorsal myeloschisis: Reconsideration of its embryological origin. Neurosurgery.

[33] Morioka T., Kanata A., Amano T., Inoue D., Suzuki S.O., Shimogawa T., Mukae N., Yoshimoto K. (2025). Saccular limited dorsal myeloschisis with a short stalk at the base of the sac causing spinal cord deviation out of the spinal canal. NMC Case Rep. J..

[34] Pang D., Dias M.S., Ahab-Barmada M. (1992). Split cord malformation: Part I: A unified theory of embryogenesis for double spinal cord malformations. Neurosurgery.

[35] Pang D., Devadass A., Thompson D. (2022). Limited dorsal myeloschisis involving one hemicord of a split cord malformation—A “hemi-LDM”. Childs Nerv. Syst..

[36] Tracy M.R., Dormans J.P., Kusumi K. (2004). Klippel-Feil syndrome: Clinical features and current understanding of etiology. Clin. Orthop. Relat. Res..

[37] Aufschnaiter K., Fellner F., Wurm G. (2008). Surgery in Adult Onset Tethered Cord Syndrome (ATCS): Review of Literature on Occasion of an Exceptional Case. Neurosurg. Rev..

[38] Pang D., Dias M.S. (1993). Cervical myelomeningoceles. Neurosurgery.

[39] Klekamp J. (2011). Tethered cord syndrome in adults. J. Neurosurg. Spine.

[40] Blount J.P., Bowman R., Dias M.S., Hopson B., Partington M.D., Rocque B.G. (2020). Neurosurgery guidelines for the care of people with spina bifida. J. Pediatr. Rehabil. Med..

