# Supplementary material for: Neglected Posterior Cervical Myelomeningocele in Adults: Systematic Review of the Literature and Illustrative Case
Source: Brain Sci. 2026 Oct 14;16(10):1107. doi: 10.3390/brainsci16101107 (PMC13702647; doi:10.3390/brainsci16101107)
Supplement: Supplementary file 1 [file brainsci-16-01107-s001.zip › brainsci-4566006-supplementary.pdf]

Neglected Posterior Cervical Myelomeningocele in Adults: Systematic Review of the Literature and Illustrative Case

Supplementary Materials

Adi Ahmetspahic, Razim Mahmutagic, Bekir Rovcanin, Ibrahim Omerhodzic, Edina Dozic, Deniz Bulja, Mirza Pojskic, Kenan I. Arnautović

Corresponding author: Mirza Pojskic, Department of Neurosurgery, Philipps University of Marburg, 35043 Marburg, Germany; mirza.pojskic@uk-gm.de

Supplementary Table S1. Database Search Strings

PubMed /MEDLINE

Strategy	Search String	Results
**A — Broad SLR (all cervical MMC)**	("Myelomeningocele"[Mesh] OR "Meningocele"[Mesh] OR myelomeningocele*[tiab] OR meningomyelocele*[tiab] OR myelocele*[tiab] OR meningocele*[tiab] OR myelocystocele*[tiab] OR "spina bifida cystica"[tiab]) AND (cervical[tiab] OR cervicothoracic[tiab] OR "cervico-thoracic"[tiab] OR cervicodorsal[tiab] OR "cervico-dorsal"[tiab] OR "cervical spine"[tiab] OR "cervical spinal"[tiab] OR "cervical cord"[tiab] OR "cervical region"[tiab])	362
**B — Posterior + Adult / Neglected presentation**	("Myelomeningocele"[Mesh] OR "Meningocele"[Mesh] OR myelomeningocele*[tiab] OR meningomyelocele*[tiab] OR myelocele*[tiab] OR meningocele*[tiab] OR myelocystocele*[tiab] OR "spina bifida cystica"[tiab]) AND (cervical[tiab] OR cervicothoracic[tiab] OR "cervico-thoracic"[tiab] OR "cervical spine"[tiab] OR "cervical spinal"[tiab] OR "cervical cord"[tiab]) AND (posterior[tiab] OR dorsal[tiab] OR "posterior cervical"[tiab] OR "dorsal cervical"[tiab] OR "posterior neck"[tiab] OR midline[tiab]) AND (adult[tiab] OR adults[tiab] OR adulthood[tiab] OR neglected[tiab] OR untreated[tiab] OR unoperated[tiab] OR unrepaired[tiab] OR "delayed presentation"[tiab] OR "late presentation"[tiab] OR "late-onset"[tiab])	25
**C — CMM + Syringomyelia**	("Myelomeningocele"[Mesh] OR "Meningocele"[Mesh] OR myelomeningocele*[tiab] OR meningomyelocele*[tiab] OR myelocele*[tiab] OR meningocele*[tiab] OR myelocystocele*[tiab] OR "spina bifida cystica"[tiab]) AND (cervical[tiab] OR cervicothoracic[tiab] OR "cervico-thoracic"[tiab] OR "cervical spine"[tiab] OR "cervical spinal"[tiab] OR "cervical cord"[tiab]) AND ("Syringomyelia"[Mesh] OR syringomyelia[tiab] OR syrinx[tiab] OR hydromyelia[tiab] OR "spinal cord cavit*"[tiab] OR "cord cavit*"[tiab] OR "intramedullary cyst*"[tiab])	62
**Date of Search**	June 4, 2026	
**Total Results**	449	

Web of Science

Strategy	Search String	Results
**A — Broad SLR (all cervical MMC)**	TS=((myelomeningocele* OR meningomyelocele* OR myelocele* OR meningocele* OR myelocystocele* OR "spina bifida cystica") AND (cervical OR cervicothoracic OR "cervico-thoracic" OR cervicodorsal OR "cervico-dorsal" OR "cervical spine" OR "cervical spinal" OR "cervical cord" OR "cervical region"))	259
**B — Posterior + Adult / Neglected presentation**	TS=((myelomeningocele* OR meningomyelocele* OR myelocele* OR meningocele* OR myelocystocele* OR "spina bifida cystica") AND (cervical OR cervicothoracic OR "cervico-thoracic" OR "cervical spine" OR "cervical spinal" OR "cervical cord") AND (posterior OR dorsal OR "posterior cervical" OR "dorsal cervical" OR "posterior neck" OR midline) AND (adult OR adults OR adulthood OR neglected OR untreated OR unoperated OR unrepaired OR "delayed presentation" OR "late presentation" OR "late-onset"))	23
**C — CMM + Syringomyelia**	TS=((myelomeningocele* OR meningomyelocele* OR myelocele* OR meningocele* OR myelocystocele* OR "spina bifida cystica") AND (cervical OR cervicothoracic OR "cervico-thoracic" OR "cervical spine" OR "cervical spinal" OR "cervical cord") AND (syringomyelia OR syrinx OR hydromyelia OR "spinal cord cavit*" OR "cord cavit*" OR "intramedullary cyst*"))	49
**Date of Search**	June 4, 2026	
**Total Results**	331	

Scopus		
**Strategy**	**Search String**	**Results**
**A — Broad SLR (all cervical MMC)**	TITLE-ABS-KEY((myelomeningocele* OR meningomyelocele* OR myelocele* OR meningocele* OR myelocystocele* OR "spina bifida cystica") AND (cervical OR cervicothoracic OR "cervico-thoracic" OR cervicodorsal OR "cervico-dorsal" OR "cervical spine" OR "cervical spinal" OR "cervical cord" OR "cervical region"))	**672**
**B — Posterior + Adult / Neglected presentation**	TITLE-ABS-KEY((myelomeningocele* OR meningomyelocele* OR myelocele* OR meningocele* OR myelocystocele* OR "spina bifida cystica") AND (cervical OR cervicothoracic OR "cervico-thoracic" OR "cervical spine" OR "cervical spinal" OR "cervical cord") AND (posterior OR dorsal OR "posterior cervical" OR "dorsal cervical" OR "posterior neck" OR midline) AND (adult OR adults OR adulthood OR neglected OR untreated OR unoperated OR unrepaired OR "delayed presentation" OR "late presentation" OR "late-onset"))	**86**
**C — CMM + Syringomyelia**	TITLE-ABS-KEY((myelomeningocele* OR meningomyelocele* OR myelocele* OR meningocele* OR myelocystocele* OR "spina bifida cystica") AND (cervical OR cervicothoracic OR "cervico-thoracic" OR "cervical spine" OR "cervical spinal" OR "cervical cord") AND (syringomyelia OR syrinx OR hydromyelia OR "spinal cord cavit*" OR "cord cavit*" OR "intramedullary cyst*"))	**112**
**Date of Search**	**September 23, 2026**	
**Total Results**	**870**	

Note: Strategies A, B, and C were run independently in each database; as B and C represent logical subsets of A (additional AND-restrictions on the core terminology), substantial overlap was expected and resolved during deduplication. Strategies B and C additionally served to characterise the proportion of the literature matching specific phenotypic profiles (posterior adult/neglected presentation; co-occurrence with syringomyelia)

Supplementary Table S2. Full Text Exclusion Log

Author (Year)	Title	Criterion	Reason for exclusion
Enjolras et al., 1995	Cervical Occult Spinal Dysraphism: MRI Findings and the Value of a Vascular Birthmark	E1	Primary diagnosis OSD with dermal sinus and dermoid cyst
Irfan et al., 2025	Tethered cervical cord syndrome: A rare entity	E1	Primary diagnosis TCS with dermal sinus tract; dorsal meningocele is secondary associated finding.
Oyar et al., 2012	Coexistence of occipital and infratorcular meningocele with cervical split cord anomaly	E1	Primary lesion is occipital/cranial meningocele; cervical split cord is associated anomaly, not primary CMM.
Singh et al., 2008	Double Meningocoele: Case Report and Anatomical Rationale	E4	Paediatric case (<18yr)
Vogter et al., 1987	High Spinal Dysrhaphism: Case Report of a Complex Cervical Meningocele	E4	Paediatric case (8 months)
Ritzel & Bonacker, 2010	Occult cervical dysraphia with tethered cord syndrome	E1	Primary diagnosis dermal sinus/occult dysraphism, not primary CMM
Akay et al., 2003 (remaining 6 patients)*	The Initial Treatment of Meningocele and Myelomeningocele Lesions in Adulthood: Experiences with Seven Patients	E1	Case series of seven adult patients; six were non-cervical (thoracic, lumbar, or sacral level) and did not meet the cervical-level inclusion criterion; only one cervical case met eligibility and was extracted
Erdinçler, 2002. (Case 1)*	Cervical cord tethering and congenital mirror movements: is it an association rather than a coincidence?	E1	Dermal sinus tract with cervicothoracic extension; wrong entity and wrong level
Wu & Scott, 1990 (Case 2)*	Myelopathy presenting decades after surgery for congenital cervical cutaneous lesions	E1	Congenital dermal sinus tract with lipoma and neurenteric cyst; wrong entity
Myung et al., 2026	Cerebrospinal fluid leak from trauma to congenital meningocele of a 40-year-old woman in Korea	E1	Meningocele at lumbosacral level, not cervical
West, 1875	Case of meningocele associated with cervical spina bifida, cured by aspiration	E4	Infant; died at 20 months
Salomão et al., 2006	Cystic spinal dysraphism of the cervical and upper thoracic region	E4	All-paediatric/neonatal series (n = 18)
Salomão, 2004	Cervical meningocele in association with spinal abnormalities	E2	Letter to the editor; no patient data reported
Fountas, 2004	Cervical meningocele in association with spinal abnormalities (reply)	E2	Letter to the editor; no patient data reported
Feltes et al., 2004	Cervical meningocele in association with spinal abnormalities	E4	Newborn
Delashaw et al., 1987	Cervical meningocele and associated spinal anomalies	E4	4 paediatric cases
Chaseling, 2006	Surgical technique and outcome in cervical and thoracic myelomeningocoeles	E2	Commentary; no patient data reported
Armas-Melián et al., 2020	Cervical myelomeningocele with CSF leakage: a case-based review	E4	Newborn
Huang, Shi & Zhang, 2010	Characteristics and surgery of cervical myelomeningocele	E4	Paediatric cases
Heidari et al., 2019	Cervical Lipomyelomeningocele Presenting with Progressive Motor Deficit	E4	Paediatric (10y); authors' diagnosis lipomyelomeningocele
Msheik et al., 2023	Cervical Myelocystocele: A One-in-50,000 Congenital Abnormality of the Spinal Cord	E4	4-month-old infant
Kaplan et al., 2008	Presence of a meningioma in a cervical myelomeningocele sac	E4	11-year-old girl
Steinbok & Cochrane, 1991	The nature of congenital posterior cervical or cervicothoracic midline cutaneous mass lesions: Report of eight cases	E4	8 paediatric cases (all neonates/infants)
Rebai et al., 2013	Mirror movements and myelomeningocele: Report of a single case and review of literature	E4	11-year-old boy
Nishio et al., 2001	Cervical (myelo)meningocoele: Report of 2 cases	E4	Both cases paediatric

Pang D, 2003	Segmental spinal cord hypoplasia and meningocele with preservation of medullary function (Commentary)	E2	Commentary; no patient data reported
Kaur et al., 2019	Cervical Lipomyelocele with Congenital Inclusion Cyst	E1	Wrong entity (authors' diagnosis cervical lipomyelocele with dermal sinus and inclusion cyst)
Flannery et al. (2003)	Delayed post-surgical development of dural arteriovenous fistula after cervical meningocele repair	E1	Dural arteriovenous fistula presenting 33 years after infantile cervical meningocele repair

Note: * partially eligible publications in which a co-reported case met all eligibility criteria and was extracted separately; E1, ineligible lesion type or level; E2, letter, reply, commentary or review without patient data; E3, insufficient detail for case-level extraction; E4, paediatric patients only (<18 years); E5, genetic, embryological or molecular findings only.

Supplementary Table S3. JBI Critical Appraisal Checklist for Case Reports

Study	Q1 Demographics	Q2 History and timeline	Q3 Presentation	Q4 Diagnostics	Q5 Intervention	Q6 Post-intervention	Q7 Adverse events	Q8 Lessons
Akay 2003 (case 1)	Y	U	Y	Y	Y	Y	Y	Y
Konya 2006	Y	Y	Y	Y	Y	Y	U	Y
Denaro 2008	Y	Y	Y	Y	Y	Y	Y	Y
Wang 2013	Y	Y	Y	Y	Y	Y	Y	Y
Raheja 2014	Y	Y	Y	Y	Y	Y	U	Y
Shamji 2015	Y	Y	Y	Y	U	Y	U	Y
Noureldine 2020	Y	Y	Y	Y	Y	Y	U	Y
Laurent 2020	Y	Y	Y	Y	Y	Y	U	Y
Mohindra 2021	Y	Y	Y	Y	Y	Y	Y	Y
Rahimizadeh 2023	Y	Y	Y	Y	Y	Y	Y	Y
Bozkurt 2024	Y	Y	Y	Y	Y	Y	Y	Y
Aribal 2016	Y	N	U	Y	NA	NA	NA	U
Chitale 2021	Y	N	Y	U	U	U	Y	U
Hepburn 1953	Y	U	Y	Y	NA	NA	NA	Y
Erdinçler 2002 (case 2)*	Y	Y	Y	Y	Y	Y	U	Y
Wu 1990 (case 1)*	Y	Y	Y	Y	Y	Y	U	Y
Lotfi 2011	Y	U	Y	Y	N	N	N	Y
Odabaşı 1998	Y	U	Y	Y	N	N	N	Y

Note: JBI scores were used descriptively to characterise methodological strengths and limitations and did not determine study eligibility *Akay et al. (2003), Erdinçler, 2002, Wu et al. (1990) were appraised using the JBI Critical Appraisal Checklist for Case Reports (8 items), as only one cervical case was extracted from this mixed-level series. Duz et al. (2008) was appraised using the JBI Critical Appraisal Checklist for Case Series (10 items; Supplementary Table S4), as all five reported cases met cervical eligibility criteria.

Q1: Were patients 'demographic characteristics clearly described?

Q2: Was the patient's history clearly described and presented as a timeline?

Q3: Was the current clinical condition of the patient on presentation clearly described?

Q4: Were diagnostic tests or assessment methods and the results clearly described?

Q5: Was the intervention(s) or treatment procedure(s) clearly described?

Q6: Was the post-intervention clinical condition clearly described?

Q7: Were adverse events (harms) or unanticipated events identified and described?

Q8: Does the case report provide takeaway lessons?

Y, yes; N, no; U, unclear; N/A, not applicable.

Supplementary Table S4. JBI Critical Appraisal Checklist for Case Series

Study	Q1 Criteria	Q2 Standard measure	Q3 Valid identification	Q4 Consecutive	Q5 Complete	Q6 Demographics	Q7 Clinical	Q8 Outcomes	Q9 Sites	Q10 Statistics
Duz 2008	U	Y	Y	U	U	Y	U	Y	U	NA

Note: JBI scores were used descriptively to characterise methodological strengths and limitations and did not determine study eligibility. Duz et al. (2008) was appraised using the JBI Critical Appraisal Checklist for Case Series (10 items). All other included studies were appraised using the JBI Critical Appraisal Checklist for Case Reports (8 items; Supplementary Table S3).

Q1: Were there clear criteria for inclusion in the case series?
Q2: Was the condition measured in a standard, reliable way for all participants?
Q3: Were valid methods used for identification of the condition for all participants?
Q4: Did the case series have consecutive inclusion of participants?
Q5: Did the case series have complete inclusion of participants?
Q6: Was there clear reporting of the demographics of the participants?
Q7: Was there clear reporting of clinical information of the participants?
Q8: Were the outcomes or follow-up results of cases clearly reported?
Q9: Was there clear reporting of the presenting sites'/clinics' demographic information?
Q10: Was statistical analysis appropriate?

Y, yes; N, no; U, unclear; N/A, not applicable.

Supplementary Table S5. Clinical, radiological, and diagnostic data of included cases (n = 24).

Study (year), country	Age/ Sex	Lesion type	Level	Prior repair	Syrinx	Presenting symptoms and examination	Objective neurological abnormality	Associated anomalies	Diagnostic findings
**Meningocele (MC), n = 16**									
Bozkurt et al., 2024, Türkiye [1]	64/ M	MC	C5–6	No	Yes	Bilateral forearm numbness, weakness and neuropathic pain (R>L), VAS 7/10. Exam: strength 4-/5 right and 4/5 left in elbow and wrist flexion, right thenar atrophy, C5–T1 hypoesthesia. Pain onset to about 3 yr earlier, meningocele diagnosed about 7 yr earlier.	Yes	C5–6 posterior arch fusion defect; floating C6 spinous process; C2–C6 syrinx	MRI: C5–6 tethering meningocele with C2–C6 syrinx; baseline IONM: normal MEP, diminished right median SSEP, absent tibial SSEP
Duz et al., 2008, Türkiye (Pt 1) [6]	22/ M	MC	C2	No	NR	Congenital midline mass with intermittent CSF leak; neurological examination normal[c]	No	C2 laminar fusion defect	MRI: C2 fusion defect; sac communicating with subdural space via stalk; cord displaced posteriorly and enlarged
Duz et al., 2008, Türkiye (Pt 2) [6]	22/ M	MC	C4–5	NR	NR	Neck pain; soft posterior cervical mass; no CSF leak or neurological deficit	No	Cervical fusion defect	MRI: meningocele extending from epidural space to skin; transverse cord tethering to posterior dura; EMG normal
Duz et al., 2008, Türkiye (Pt 3) [6]	20/ M	MC	C3–4	NR	NR	Neck pain; posterior cervical mass. Exam: left upper-limb weakness, mainly of the fingers; hypoactive biceps reflexes; no sensory impairment in the case text (abstract: left-hand hypesthesia).	Yes	C3–4 laminar fusion defect	CT/MRI: C3–4 fusion defect; solid mass communicating with epidural space; cord not widened
Duz et al., 2008, Türkiye (Pt 4) [6]	20/ M	MC	C6–7	NR	NR	Neck pain; posterior cervical mass; no CSF leak or neurological deficit	No	Small vertebral bodies at C7/T1[e]	MRI: meningocele from C6 to skin; transverse cord tethering; EMG normal
Duz et al., 2008, Türkiye (Pt 5) [6]	18/ M[f]	MC	C6–7	NR	NR	Pain-causing cervical mass. Exam: hypoactive biceps and triceps reflexes.	Yes	Cervical fusion defects; type 2 split cord malformation C5–7; Klippel–Feil syndrome	MRI: C6–7 meningocele; transverse cord tethering; SCM type 2; fused C6–T1 vertebral bodies
Konya et al., 2006, Türkiye [7]	47/ M	MC	C4–5	No	NR	Leakage of colourless fluid from sac (3 mo); gait difficulty and lower-limb stiffness. Exam: hyperreflexia, bilateral Babinski sign.	Yes	C4–5 laminar dysplasia; transverse cord tethering	MRI: sac communicating with epidural/subarachnoid spaces; tethered cord; CT: defective C4–5

									laminae; abnormal evoked potentials and chronic C6–7 impairment
Akay et al., 2003, Türkiye [16]	21/M	MC	C2–3	No	No	Asymptomatic cervical lesion; surgery for cosmetic reasons; neurological examination normal.	No	C2–3 spina bifida; cord tethering	X-ray/CT/MRI: cervical meningocele and C2–3 spina bifida; no hydromyelia; EMG/SSEP normal
Shamji & Ibrahim, 2015, Canada [17]	22/F	MC	NR	NR	NR	Congenital posterior cervical lesion, enlarging over years; no leakage; no meningitis or myeloradiculopathy. Exam: bilateral Hoffmann sign and UMN signs in both upper limbs.	Yes	Dorsal cord tethering with connecting tract	MRI: fluid-filled dorsal cutaneous lesion with dorsal cord tethering
Hepburn, 1953, UK [26]	25/M	Presumed MC	C2	NR	NR	Behaviour disorder, head and neck pain, vomiting, occipital headache, drowsiness; transient suboccipital swelling. Exam: widely dilated, sluggish pupils; vertical nystagmus; normal reflexes.	NR	Bifid hypertrophied C2; meninges-lined channel communicating with canal	No antemortem imaging; autopsy showed C2 bifid spine and meninges-lined channel
Erdinçler., 2002, Türkiye (Case 2) [27]	18/F	MC, repaired	C2–3	Yes	NR	Progressive gait disturbance; mirror movements of the hands. Exam: hyperactive reflexes, bilateral Babinski signs, broad-based unsteady gait; no sensory deficit.	Yes	Cerebellar tonsil displacement to C2; mild ventricular dilatation; spina bifida; cord tethering	MRI: C2–3 deformity/tethering and central canal dilatation; abnormal EMG/TMS/SEP
Wu & Scott, 1990, USA (Case 1) [28]	43/M	Probable MC	C3–4	Yes	Yes	Progressive loss of fine hand motor skills; multiple sensory deficits and weakness	Yes	Dorsal fibrous tract with aberrant roots; cord tethering	MRI: C3–4 dorsal soft-tissue tract with small intramedullary cavity, consistent with tethering/syringomyelia
Lotfi et al., 2011, Iran [29]	24/M	MC	Upper cervical	NR	NR	Complex partial seizures; congenital posterior neck mass; neurological examination normal.	No	Bilateral subependymal nodular heterotopia	MRI: posterior cervical meningocele with intact skin; brain MRI showed bilateral subependymal nodular heterotopia; no Chiari
Odabaşı et al., 1998, Türkiye [30]	20/M	MC	Suboccipital	NR	NR	Mirror movements; midline suboccipital mass; other neurological findings normal.	Yes	Anomaly posterior to cord–medulla junction	MRI: cervical meningocele; CT: no connection with subarachnoid space; abnormal motor but not sensory pathways
Rahimizadeh et al., 2023, Iran [3]	34/M	Rudimentary MC	C4–5	No	NR	Congenital skin-covered solid posterior cervical mass; cosmetic excision; neurological examination normal	No	C4–5 elongated spinous processes/bifid laminae; limited cord split	MRI: solid mass with fibrous stalk to dorsal cord; C4–5 spina bifida; limited cord split
Wang et al., 2013, China [19]	21/F	Rudimentary MC	C3	NR	NR	Congenital enlarging posterior cervical mass; cosmetic excision; “hair collar” sign; no neurological deficit	No	C3 laminar fusion defect; dorsal cord tenting; central canal enlargement; small arachnoid cyst[k]	CT/MRI: C3 fusion defect; solid mass connected to dorsal cord by stalk; central canal enlargement and small cyst

**Myelocystocele (MyCC), n = 1**									
Raheja et al., 2014, India [20]	42/ M	Non-terminal MyCC	C6–7	NR	Yes	Progressive spastic quadriparesis, sensory loss and bowel/bladder dysfunction	Yes	Arrested hydrocephalus; mild Chiari II; C6/7 and T1/2 fusion; C6–7 spina bifida; open-book cord[p]	MRI: non-terminal myelocystocele with cord tenting and ependymal-lined cyst continuous with hydromyelic cavity; cranial syrinx
**Myelomeningocele (MMC), n = 7 (six previously reported cases and the present case)**									
Denaro et al., 2008, Italy [5]	52/ M	MMC	C4–5	No	Yes	Progressive bilateral upper-limb weakness and paresthesia (3 yr); posterior neck mass. Exam: slight increase of upper-limb deep tendon reflexes.	Yes	Diplomyelia; cervical spina bifida; syrinx; posterior cord tethering	MRI: complex cervical dysraphism with cord tethering and cranial/caudal syrinx; SSEP/EMG normal
Noureldine et al., 2020, USA [8]	47/ M	Recurrent MMC	C4–6	Yes	No	Progressive neck/shoulder pain, impaired finger dexterity and gait instability. Exam: mild bilateral handgrip weakness; numbness and diminished proprioception in fingers and lower limbs; impaired tandem gait.	Yes	Absent C5 spinous process; cord, nerve roots and CSF within sac; dorsal cord tethering	MRI: C4–6 posterior bulge with absent C5 spinous process and neural elements within sac
Laurent et al., 2020, USA [9]	35/ M	Retethered MMC	C4–5	Yes[s]	Yes	Progressive left upper-limb weakness, numbness and hand clumsiness. Exam: left hand intrinsic strength 4-/5, decreased light touch in the left hand, left upper-limb hyperreflexia.	Yes	Posterior element defect; neural elements projecting subcutaneously; lumbar diastematomyelia with low-lying conus	MRI: C4–5 cord expansion/T2 hyperintensity, posterior neural projection and small syrinx
Mohindra et al., 2021, India [18]	18/ M	MMC with malignant transformati on	C3	No	NR	Congenital swelling; adolescent spastic paraparesis (Nurick grade 4); recent rapid enlargement, CSF leak and ulceration	Yes	C3 spinous process defect; cervical cord tethering	MRI/CT: large dorsal cervical cyst with CSF-like fluid communicating through C3 defect; cord tethering
Aribal et al., 2016, Türkiye [24]	28/F	MMC	C6	NR	Yes	Chronic cervical/dorsal pain and sensory loss	NR	Multilevel posterior fusion defects; C2–3/C5–6 vertebral fusion; Chiari; kyphoscoliosis; syringohydromyelia	MRI: cord/dural herniation through C6 defect; large syringohydromyelia; Chiari and segmentation anomalies
Chitale & Deshmukh, 2021, India [25]	31/ M	MMC	C5–7	NR	NR	Upper-limb/cervical pain and numbness; fatty cervical lump. Exam: decreased muscle tone	Yes	Cervical vertebral wedging[h]	MRI: abnormal fatty mass at C5–7 with cervical tethering; vertebral wedging
Present case, Bosnia and Herzegovina	***62/ M***	***Radiological ly suspected MMC***	***C4–5***	***No***	***No***	***Intermittent left-hand numbness during prolonged driving; congenital posterior cervical lesion; no persistent myelopathic symptoms. Neurological examination normal.***	***No***	***C4–5 block vertebra/posterior defect; split cord malformation; supernumerary C7 rib; cervical scoliosis; ventriculomegaly/arrested hydrocephalus***	***CT/MRI: dorsal herniation of dural sac/neural elements; cord tethering/tenting; split cord malformation; no syringohydromyelia***

Note: Cases are grouped by the lesion type given by the original authors (no reclassification) and ordered as in Table 2, with the present case last (bold/italic). Abbreviations: ACM II, Arnold–Chiari malformation type II; CSF, cerebrospinal fluid; CT, computed tomography; EMG, electromyography; IONM, intraoperative neuromonitoring; MC, meningocele; MEP, motor evoked potentials; MMC, myelomeningocele; MRI, magnetic resonance imaging; MyCC, myelocystocele; mo, months; N/A, not applicable (documented absence of intervention); NR, not reported; Pt, patient; SCM, split cord malformation; SEP, somatosensory evoked potentials; SSEP, somatosensory evoked potentials; TMS, transcranial magnetic stimulation; UMN, upper motor neuron; VAS, visual analogue scale; yr, years.

Supplementary Table S6. Operative management, histopathology, follow-up, and outcome of included cases (n = 24).

Study (year), country	Indication for surgery	Surgical management	Histopathology	Follow-up (months)	Outcome	Adverse events	Recurrence or progression at last follow-up	Outcome category
**Meningocele (MC), n = 16**								
Bozkurt et al., 2024, Türkiye [1]	Symptomatic tethering meningocele with syrinx; failed prior treatment	C4–C6 laminectomy, intradural adhesiolysis, meningocele excision, stalk cauterization, detethering, watertight closure	No neural tissue; meningothelial cells and psammoma bodies	12 (also 6)	Improved; cord untethered, syrinx significantly resolved; residual left-arm tingling	Yes (transient, intraoperatie): MEP fall to 10% of baseline on neck flexion, resolved within 5 min; no additional deficit after extubation	NR	IMP
Duz et al., 2008, Türkiye (Pt 1) [6]	CSF leak and cyst resection	Microscopic/circumferential resection, stalk division, intradural exploration, release of fibrous tethering bands, dural repair	Meningothelial heterotopia with psammoma bodies; no neural elements	12	Neurologically intact; tethering and cord enlargement resolved; no symptom recurrence	None (series statement)	N	INT
Duz et al., 2008, Türkiye (Pt 2) [6]	Pain relief and cyst resection	Lesion excision, partial laminectomy, release of tethering bands, neck ligation	NR (no patient-specific description; series statement: no neural elements)	12	Neurologically intact; no neurological disturbance at 1 yr	None (series statement)	NR	INT
Duz et al., 2008, Türkiye (Pt 3) [6]	Neurological deficit, pain relief and cyst resection	Partial laminectomy, sac resection, release of dural attachments	No neural/neoplastic components; meningocele	12	Finger weakness resolved; no new deficit or symptom recurrence	None (series statement)	N	IMP
Duz et al., 2008, Türkiye (Pt 4) [6]	Pain relief and cyst resection	Circumferential dissection, release of tethering attachments, mass/stalk excision	NR (no patient-specific description; series statement: no neural elements)	12	Neurologically intact; no neurological disturbance at 1 yr	None (series statement)	NR	INT
Duz et al., 2008, Türkiye (Pt 5) [6]	Pain relief and cyst resection	Meningocele excision, release of cord attachments, duraplasty; SCM fibrous band left untouched	No neural/neoplastic components; cervical meningocele	24	No symptom recurrence or neurological deterioration over 2 yr	None (series statement)	N	STB
Konya et al., 2006, Türkiye [7]	CSF leakage and upper motor neuron deficits	Partial laminectomy, sac excision, release of fibrovascular/arachnoid bands, duraplasty	No neural/neoplastic components; cervical meningocele	12	Walking difficulty and leg stiffness resolved; no symptom recurrence	NR (only neurological status on awakening reported)	N	IMP

Akay et al., 2003, Türkiye [16]	Cosmetic reasons; cord untethering	Excision, laminectomy, intradural release of lesion–cord connections, watertight closure	NR	24	Neurologically intact and asymptomatic	Yes (intraoperative): fecal incontinence during circumferential dissection, postoperative course uneventful	NR	INT
Shamji & Ibrahim, 2015, Canada [17]	Enlarging lesion with UMN signs	Laminectomy, fibrous stalk resection, cord detethering, expansile duraplasty	NR	NR	No neurological deterioration or CSF leak; no visible recurrence at last follow-up	NR	N	STB
Hepburn, 1953, UK [26]	N/A	No treatment	NR for cervical lesion	N/A	Died 10 days after admission; autopsy: bronchial carcinoma with multiple cerebral metastases	N/A	N/A	N/A
Erdinçler, 2002, Türkiye (Case 2) [27]	Untethering and craniocervical decompression	Posterior fossa craniectomy, C1–2 laminectomy, intradural release of scar/arachnoid bands, duraplasty	NR	NR	Gait gradually improved; mirror movements persisted	NR	NR	IMP
Wu & Scott, 1990, USA (Case 1) [28]	Symptoms attributed to tethering	C2–5 laminectomy; resection of aberrant roots tethering the cord; syrinx drainage	NR	6	Excellent recovery; returned to work; improved sensation and strength with residual left hand weakness	NR	NR	IMP
Lotfi et al., 2011, Iran [29]	NR	NR	NR	NR	NR	NR	NR	NR
Odabaşı et al., 1998, Türkiye [30]	NR	NR	NR	NR	NR	NR	NR	NR
Rahimizadeh et al., 2023, Iran [3]	Cosmetic excision	Mass excision, C4–5 laminectomy, stalk dissection/detethering, dural closure	Meningothelial cells and psammoma bodies; EMA/PR positive	NR	Uneventful recovery; postoperative neurological status/follow-up not reported	None	NR	NR
Wang et al., 2013, China [19]	Cosmetic excision	C3 laminectomy, stalk resection, intradural cyst fenestration, tethering-band release, dural repair	Meningocyte whorls/psammoma bodies; no neural elements in mass; dysplastic glia/neurons in tethering band	12 (MRI at 6)	Uneventful; MRI showed removal and no further tethering; no neurological deficit	None	N	INT

**Myelocystocele (MyCC), n = 1**								
Raheja et al., 2014, India [20]	Progressive spastic quadriparesis, sensory and bowel/bladder dysfunction	Complete excision of tethering band; intraspinal adhesiolysis	Glial and adipose tissue in fibrocollagenous background, confirming myelocystocele	6 (MRI at 3)	Reduced spasticity and improved power; stable at 6 mo; syrinx/Chiari II non-progressive	NR	N	IMP
**Myelomeningocele (MMC), n = 7 (six previously reported cases and the present case)**								
Denaro et al., 2008, Italy [5]	Progressive adult symptoms	Sac resection, intradural exploration, stalk transection/excision, detethering	Meningothelial cells/psammoma bodies; rare glial tissue	6	Early neurological improvement; asymptomatic at 6 mo; syrinx shrinking on MRI	None	NR	IMP
Noureldine et al., 2020, USA [8]	Prevent symptom progression	Neurophysiological monitoring; C4–T1 exposure; placode/roots released; MMC reduced into dura and closed	NR	1	No new deficit; neck pain resolved; improved proprioception and gait	NR	NR	IMP
Laurent et al., 2020, USA [9]	Symptomatic retethered MMC	Laminectomy, intradural detethering, division of arachnoid bands, resection of dysplastic cord projection, watertight closure	Neuroglial tissue/meninges with fibrosis and disorganized tissue	4 (also 1)	Progressive improvement; full hand strength at 4 mo; MRI confirmed successful detethering	NR	NR	IMP
Mohindra et al., 2021, India [18]	Rapid enlargement, CSF leak, ulceration, spastic paraparesis	C2–4 laminectomy, CMMC excision, cord/root detethering, sac excision, watertight closure; radiotherapy	Pleomorphic leiomyosarcoma arising in meningocele; tumour-free margins	12 (MRI at 9)	Uneventful; MRI at 9 mo showed untethered cord; "asymptomatic" at 1 yr per source table	None	NR	IMP
Aribal et al., 2016, Türkiye [24]	N/A; surgery declined	No surgery; conservative follow-up	N/A	NR	No further outcome reported	N/A	NR	N/A
Chitale & Deshmukh, 2021, India [25]	Pain/numbness and reduced tone	MMC correction (operative details not reported)	NR	NR	No postoperative complications; neurological outcome not reported	None	NR	NR
Present case, Bosnia and Herzegovina	***N/A; observation (no deficit, progression, CSF leakage or local complications); surgery declined***	***N/A; Observation with clinical/radiological follow-up***	***N/A***	***3***	***Neurologically intact; no new deficit or symptom progression***	***N/A***	***N***	***N/A***

Note: Cases are grouped by the lesion type given by the original authors (no reclassification) and ordered as in Table 2, with the present case last (bold/italic). Outcome category (authors' wording): IMP, improvement of a preoperative deficit or symptom; INT, neurologically intact before and after treatment; STB, no deterioration or recurrence without stated improvement. Abbreviations: CMMC, cervical myelomeningocele (authors' abbreviation); CSF, cerebrospinal fluid; EMA, epithelial membrane antigen; IMP, improved; INT, neurologically intact; MEP, motor evoked potentials; MMC, myelomeningocele; MRI, magnetic resonance imaging; mo, months; N/A, not applicable NR, not reported; PR, progesterone receptor; Pt, patient; SCM, split cord malformation; STB, stable; UMN, upper motor neuron; yr, years.
