# Supplementary material for: Neglected Posterior Cervical Myelomeningocele in Adults: Systematic Review of the Literature and Illustrative Case
Source: Brain Sci. 2026 Oct 14;16(10):1107. doi: 10.3390/brainsci16101107 (PMC13702647; doi:10.3390/brainsci16101107)
Supplement: Supplementary file 1 [file brainsci-16-01107-s001.zip › PRISMA_2020_Checklist.pdf]

PRISMA 2020 Checklist

Section and Topic	Item #	Checklist item	Location where item is reported
**TITLE**			
Title	1	Identify the report as a systematic review.	Title page ("Neglected Posterior Cervical Myelomeningocele in Adults: Systematic Review of the Literature and Illustrative Case")
**ABSTRACT**			
Abstract	2	See the PRISMA 2020 for Abstracts checklist.	Abstract (Background, Methods, Results, Conclusions)
**INTRODUCTION**			
Rationale	3	Describe the rationale for the review in the context of existing knowledge.	Introduction, paragraph 3 ("Although Ricci et al. addressed anterior cervical meningocele in adults and Maroufi et al. reviewed saccular limited dorsal myeloschisis, previously called cervical MMC, in predominantly paediatric patients, no equivalent synthesis exists for posterior cervical spinal dysraphism in the adult population.")
Objectives	4	Provide an explicit statement of the objective(s) or question(s) the review addresses.	Introduction, paragraph 3. ("The objective of this study was to conduct, to our knowledge, the first systematic review of posterior cervical spinal dysraphism in adults.")

PRISMA 2020 Checklist

Section and Topic	Item #	Checklist item	Location where item is reported
**METHODS**			
Eligibility criteria	5	Specify the inclusion and exclusion criteria for the review and how studies were grouped for the syntheses.	Methods, Section 2.2.2 (eligibility criteria; grouping by the label used in the original report into MC, MyCC and MMC); Table 1
Information sources	6	Specify all databases, registers, websites, organisations, reference lists and other sources searched or consulted to identify studies. Specify the date when each source was last searched or consulted.	Methods, Section 2.2.1 (MEDLINE via PubMed, 4 June 2026; Web of Science Core Collection, 4 June 2026; Scopus, 23 September 2026; backward and forward citation searching; Embase not searched; no other sources searched)
Search strategy	7	Present the full search strategies for all databases, registers and websites, including any filters and limits used.	Methods, Section 2.2.1; full search strings for Strategies A, B and C for each database, including Scopus, in Supplementary Table S1; no date or language restrictions
Selection process	8	Specify the methods used to decide whether a study met the inclusion criteria of the review, including how many reviewers screened each record and each report retrieved, whether they worked independently, and if applicable, details of automation tools used in the process.	Methods, Section 2.2.2 (two independent reviewers, R.M. and B.R.; disagreements resolved by a third reviewer, A.A.); Section 2.2.1 (deduplication with ASySD; screening in Rayyan)
Data collection process	9	Specify the methods used to collect data from reports, including how many reviewers collected data from each report, whether they worked independently, any processes for obtaining or confirming data from study investigators, and if applicable, details of automation	Methods, Section 2.2.4 "Data

Section and Topic	Item #	Checklist item	Location where item is reported
		tools used in the process.	Extraction and Synthesis" (two reviewers independently; third reviewer for disagreements); study investigators were not contacted
Data items	10a	List and define all outcomes for which data were sought. Specify whether all results that were compatible with each outcome domain in each study were sought (e.g. for all measures, time points, analyses), and if not, the methods used to decide which results to collect.	Methods, Section 2.2.4 (list of extracted variables)
	10b	List and define all other variables for which data were sought (e.g. participant and intervention characteristics, funding sources). Describe any assumptions made about any missing or unclear information.	Methods, Section 2.2.4 (country of origin, demographics and other variables listed; unreported data coded NR; N/A only for a documented absence of intervention; denominator stated for subsets)
Study risk of bias assessment	11	Specify the methods used to assess risk of bias in the included studies, including details of the tool(s) used, how many reviewers assessed each study and whether they worked independently, and if applicable, details of automation tools used in the process.	Methods, Section 2.2.3 "Methodological Appraisal (JBI)" (JBI checklists used descriptively as critical appraisal tools, item by item, without a summed score or risk-of-bias category); Section 2.2.4 (two reviewers independently); Supplementary Tables S3 and S4
Effect measures	12	Specify for each outcome the effect measure(s) (e.g. risk ratio, mean difference) used in the synthesis or presentation of results.	Not applicable; no meta-analysis performed; narrative synthesis only (Methods 2.2.4)
Synthesis	13a	Describe the processes used to decide which studies were eligible for each synthesis (e.g. tabulating the study intervention	Methods, Section

Section and Topic	Item #	Checklist item	Location where item is reported
methods		characteristics and comparing against the planned groups for each synthesis (item #5)).	2.2.2 (grouping by original label) and Section 2.2.4 (narrative synthesis by lesion group)
	13b	Describe any methods required to prepare the data for presentation or synthesis, such as handling of missing summary statistics, or data conversions.	Not applicable
	13c	Describe any methods used to tabulate or visually display results of individual studies and syntheses.	Table 2; Table 3 (item-level JBI ratings); Table 4 (characteristics by lesion group); Supplementary Tables S5 and S6
	13d	Describe any methods used to synthesize results and provide a rationale for the choice(s). If meta-analysis was performed, describe the model(s), method(s) to identify the presence and extent of statistical heterogeneity, and software package(s) used.	Methods, Section 2.2.4 (narrative synthesis presented in tables by lesion group; meta-analysis not performed because the evidence consists of case reports and small series)
	13e	Describe any methods used to explore possible causes of heterogeneity among study results (e.g. subgroup analysis, meta-regression).	No formal exploration of heterogeneity; presentation and age were described narratively and by lesion group (Results 3.2.3; Table 4; Discussion 4.2)
	13f	Describe any sensitivity analyses conducted to assess robustness of the synthesized results.	Not applicable
Reporting bias assessment	14	Describe any methods used to assess risk of bias due to missing results in a synthesis (arising from reporting biases).	Methods, Section 2.2.4 (publication bias not assessed formally because the evidence consists of case reports and small series); discussed in Discussion, Section

Section and Topic	Item #	Checklist item	Location where item is reported
			4.4
Certainty assessment	15	Describe any methods used to assess certainty (or confidence) in the body of evidence for an outcome.	Not applicable
**RESULTS**			
Study selection	16a	Describe the results of the search and selection process, from the number of records identified in the search to the number of studies included in the review, ideally using a flow diagram.	Results, Section 3.2.1 "Study Selection"; Figure 1 (PRISMA flow diagram) shown in Section 2.2.2
	16b	Cite studies that might appear to meet the inclusion criteria, but which were excluded, and explain why they were excluded.	Results, Section 3.2.1; excluded full-text reports and reasons in Supplementary Table S2
Study characteristics	17	Cite each included study and present its characteristics.	Results, Section 3.2.1 (included studies and countries) and Section 3.2.3 (characteristics, management and outcomes); Table 2; Table 4; Supplementary Tables S5 and S6
Risk of bias in studies	18	Present assessments of risk of bias for each included study.	Results, Section 3.2.2 "Quality Assessment"; Table 3 (item-level JBI ratings, critical appraisal and not risk of bias); Supplementary Tables S3 and S4
Results of individual studies	19	For all outcomes, present, for each study: (a) summary statistics for each group (where appropriate) and (b) an effect estimate and its precision (e.g. confidence/credible interval), ideally using structured tables or plots.	Table 2; Supplementary Tables S5 and S6
Results of syntheses	20a	For each synthesis, briefly summarise the characteristics and risk of bias among contributing studies.	Results, Sections 3.2.2 and 3.2.3; Table 4
	20b	Present results of all statistical syntheses conducted. If meta-analysis was done, present for each the summary estimate and its precision (e.g. confidence/credible interval) and measures of statistical heterogeneity. If comparing groups, describe the direction of the	Not applicable

Section and Topic	Item #	Checklist item	Location where item is reported
		effect.	
	20c	Present results of all investigations of possible causes of heterogeneity among study results.	Not applicable
	20d	Present results of all sensitivity analyses conducted to assess the robustness of the synthesized results.	Not applicable
Reporting biases	21	Present assessments of risk of bias due to missing results (arising from reporting biases) for each synthesis assessed.	Discussion, Section 4.4 (Limitations)
Certainty of evidence	22	Present assessments of certainty (or confidence) in the body of evidence for each outcome assessed.	Not applicable
**DISCUSSION**			
Discussion	23a	Provide a general interpretation of the results in the context of other evidence.	Discussion, Section 4; and Sections 4.1–4.3
	23b	Discuss any limitations of the evidence included in the review.	Discussion, Section 4.4 "Limitations and Future Directions"
	23c	Discuss any limitations of the review processes used.	Discussion, Section 4.4 (Embase not searched; no sources other than citation searching; six reports not retrieved; protocol amended twice after the initial searches; publication bias not assessed formally; JBI appraisal descriptive only)
	23d	Discuss implications of the results for practice, policy, and future research.	Discussion, Sections 4.3 and 4.4 "Limitations and Future Directions"; Section 5
**OTHER INFORMATION**			
Registration and protocol	24a	Provide registration information for the review, including register name and registration number, or state that the review was not registered.	Methods, Section 2.2.1 (PROSPERO registration CRD420261426944)
	24b	Indicate where the review protocol can be accessed, or state that a protocol was not prepared.	PROSPERO (registration CRD420261426944)
	24c	Describe and explain any amendments to information provided at registration or in the protocol.	Methods, Section 2.2.5 "Protocol

PRISMA 2020 Checklist

Section and Topic	Item #	Checklist item	Location where item is reported
			Amendments"
Support	25	Describe sources of financial or non-financial support for the review, and the role of the funders or sponsors in the review.	Funding
Competing interests	26	Declare any competing interests of review authors.	Conflicts of Interest
Availability of data, code and other materials	27	Report which of the following are publicly available and where they can be found: template data collection forms; data extracted from included studies; data used for all analyses; analytic code; any other materials used in the review.	Data Availability Statement

From: Page MJ, McKenzie JE, Bossuyt PM, Boutron I, Hoffmann TC, Mulrow CD, et al. The PRISMA 2020 statement: an updated guideline for reporting systematic reviews. BMJ 2021;372:n71. doi: 10.1136/bmj.n71
